# GSK3β Impairs KIF1A Transport in a Cellular Model of Alzheimer’s Disease but Does Not Regulate Motor Motility at S402

**DOI:** 10.1523/ENEURO.0176-20.2020

**Published:** 2020-11-04

**Authors:** K.J. Gan, A. Akram, T.L. Blasius, E.M. Ramser, B.G. Budaitis, D.R. Gabrych, K.J. Verhey, M.A. Silverman

**Affiliations:** 1 Department of Molecular Biology and Biochemistry, Simon Fraser University, Burnaby, BC, V5A1S6; 2 Department of Biological Sciences, Simon Fraser University, Burnaby, BC, V5A1S6; 3 Centre for Cell Biology, Development and Disease, Simon Fraser University, Burnaby, BC, V5A1S6; 4 Department of Cell and Developmental Biology, University of Michigan Medical School; 5 Program in Cellular and Molecular Biology, University of Michigan, Ann Arbor, Michigan, 94305

**Keywords:** Alzheimer’s disease, amyloid beta oligomers, axonal transport, glycogen synthase kinase beta, kinesin-3 (KIF1A), motor protein phosphorylation

## Abstract

Impairment of axonal transport is an early pathologic event that precedes neurotoxicity in Alzheimer’s disease (AD). Soluble amyloid-β oligomers (AβOs), a causative agent of AD, activate intracellular signaling cascades that trigger phosphorylation of many target proteins, including tau, resulting in microtubule destabilization and transport impairment. Here, we investigated how KIF1A, a kinesin-3 family motor protein required for the transport of neurotrophic factors, is impaired in mouse hippocampal neurons treated with AβOs. By live cell imaging, we observed that AβOs inhibit transport of KIF1A-GFP similarly in wild-type and tau knock-out neurons, indicating that tau is not required for this effect. Pharmacological inhibition of glycogen synthase kinase 3β (GSK3β), a kinase overactivated in AD, prevented the transport defects. By mass spectrometry on KIF1A immunoprecipitated from transgenic AD mouse brain, we detected phosphorylation at S402, which conforms to a highly conserved GSK3β consensus site. We confirmed that this site is phosphorylated by GSK3β *in vitro*. Finally, we tested whether a phosphomimic of S402 could modulate KIF1A motility in control and AβO-treated mouse neurons and in a Golgi dispersion assay devoid of endogenous KIF1A. In both systems, transport driven by mutant motors was similar to that of WT motors. In conclusion, GSK3β impairs KIF1A transport but does not regulate motor motility at S402. Further studies are required to determine the specific phosphorylation sites on KIF1A that regulate its cargo binding and/or motility in physiological and disease states.

## Significance Statement

Axonal transport of proteins and organelles is required for neuronal function and survival and is impaired in Alzheimer’s disease (AD). Pathogenic mechanisms that directly impact motor protein motility before neuronal toxicity have not been widely investigated. Here, we show that KIF1A, the primary kinesin motor required for transport of neurotrophic factors, is impaired in mouse neurons treated with amyloid-β oligomers (AβOs), a causative agent of AD. Inhibition of glycogen synthase kinase 3β (GSK3β), a kinase overactivated in AD, prevents these defects. We detected phosphorylation of S402, a highly conserved GSK3β consensus site, in KIF1A immunoprecipitated from AD mouse brain. However, a phosphomimic of S402 did not modulate KIF1A motility in cell-based assays. Thus, GSK3β impairs KIF1A transport but likely not through phosphorylation at S402.

## Introduction

Neurons rely on microtubule-based, fast axonal transport of proteins and organelles for development, communication, and survival. Transport is driven by kinesin and cytoplasmic dynein motor proteins that carry cargoes anterogradely toward the synapse or retrogradely toward the cell body, respectively. Impaired motor-cargo binding and/or disrupted motor-microtubule interactions are common to several neurodegenerative diseases (Brady and Morfini, 2017). Importantly, they arise before neurite dystrophy, synapse loss, and cell death. In experimental models of Alzheimer’s disease (AD), axonal transport of neuropeptide vesicles and mitochondria are perturbed by soluble amyloid-β oligomers (AβOs; Decker et al., 2010; Gan and Silverman, 2015; Seifert et al., 2016; Zhang et al., 2018), which accumulate in AD brain before detectable formation of Aβ plaques and neurofibrillary tangles (Ferreira et al., 2015). AβOs activate intracellular signaling cascades that lead to phosphorylation and aberrant activation of many target proteins, including the microtubule-associated protein tau (Cline et al., 2018). Although much research has focused on tau hyperphosphorylation and on subsequent microtubule destabilization and cytoskeletal collapse that halt transport in advanced stages of AD, it fails to account for earlier, subtler and potentially reversible mechanisms that modulate transport independent of tau. Thus, we focused our investigation on AβO-activated kinases that might directly impair motor protein activity.

AD-associated kinases phosphorylate kinesin-I (KIF5) and thereby impair motility and cargo binding (Gibbs et al., 2015; Brady and Morfini, 2017); however, it is unknown whether they regulate the kinesin-3 motor, KIF1A. KIF1A associates with several different membranous organelles such as dense core vesicles (DCVs), which contain critical signaling molecules including brain-derived neurotrophic factor (BDNF; Yonekawa et al., 1998; Lo et al., 2011; Kondo et al., 2012; Hung and Coleman, 2016). Despite the importance of BDNF in neuronal physiology and its reduced availability in AD and other neurodegenerative diseases (Mariga et al., 2017), mechanisms that regulate its transport by KIF1A are unclear. Previously, Decker et al. (2010) demonstrated that AβOs impair axonal BDNF transport in primary hippocampal neurons. Contrary to a current model (Bloom, 2014), these transport defects are induced independent of tau, microtubule destabilization, and acute cell death (Ramser et al., 2013). Furthermore, in these studies, BDNF transport impairment was prevented by inhibiting glycogen synthase kinase 3β (GSK3β), a kinase that is implicated in many aspects of AD pathogenesis (Maqbool et al., 2016) and regulates kinesin-1 interactions with cargo and microtubules (Morfini et al., 2002; Weaver et al., 2013). Whether AβO-induced dysregulation of GSK3β signaling directly affects KIF1A motility has not been investigated.

Here, we show by live cell imaging that AβOs impair KIF1A motility in wild-type (WT) and tau knock-out hippocampal neurons, indicating that tau is not required for KIF1A transport disruption. Pharmacological inhibition of GSK3β prevents these defects, and GSK3β coimmunoprecipitates with KIF1A, indicating that this kinase may regulate motility. By mass spectrometry (MS) on KIF1A isolated from AD mouse brain, we identified several phosphopeptides targeted by kinases associated with AD, including GSK3β. We discovered that S402, located proximal to the neck-coil domain, conforms to a highly conserved GSK3β consensus site and hypothesized that it might regulate KIF1A processivity. Thus, we tested whether a phosphomimic of S402 could modulate KIF1A motility in mouse neurons and in a Golgi dispersion assay devoid of endogenous KIF1A (Engelke et al., 2016; Budaitis et al., 2019). In both systems, we did not observe any alterations in the motility of KIF1A. Further studies will be required to determine whether other phosphorylation sites on KIF1A regulate its motility or binding to DCVs in physiological and AD states.

## Materials and Methods

### Hippocampal cell culture and expression of transgenes

Primary hippocampal neurons from embryonic day (E)16 WT and tau knock-out mice of either sex (The Jackson Laboratory) were cultured as described previously (Kaech and Banker, 2006) and kept in PNGM primary neuron growth media (Lonza). The glial feeder layer was derived from murine neural stem cells as described previously (Miranda et al., 2012). At 10–12 d *in vitro* (DIV), cells were cotransfected using Lipofectamine 2000 (Invitrogen) with plasmids encoding soluble blue fluorescent protein (pmUβA-eBFP) and mouse KIF1A-GFP (GW1-KIF1A-eGFP; Lee et al., 2003). Cells expressed constructs for 36 h before live imaging of KIF1A transport. All experiments with animals were approved by and followed the guidelines set out by the University Animal Care Committee, Protocol 1261B-05.

### AβO and GSK3β Inhibitor VIII treatments

Soluble, full-length Aβ 1–42 peptides (American Peptide) were prepared exactly according to the method of Lambert et al., 2007 (Lambert et al., 2007) and applied to cells at a final concentration of 500 nm for 18 h. Cells were incubated with 5 μm GSK3β Inhibitor VIII (Calbiochem) or equivalent volumes of vehicle (EtOH) 30 min before AβO or vehicle treatment.

### Live imaging and analysis of KIF1A transport

KIF1A-GFP transport was analyzed using a standard wide-field fluorescence microscope equipped with a cooled charge-coupled device camera and controlled by MetaMorph (Molecular Devices) as described previously (Kwinter and Silverman, 2009; Gan and Silverman, 2016). All imaging, typically 100 frames, was recorded by the “stream acquisition module” in MetaMorph. Briefly, cells were sealed in a heated imaging chamber, and recordings were acquired from double transfectants at an exposure time of 250 ms for 90 s. This captured dozens of transport events per cell in 100-μm segments of the axon. Dendrites and axons were initially identified based on morphology and confirmed retrospectively by immunostaining against MAP2, a dendritic cytoskeletal protein. Soluble BFP detection was necessary to determine the orientation of the cell body relative to the axon and thus to distinguish between anterograde and retrograde transport events. Motor protein flux, velocity, and run lengths were obtained through tracing kymographs in MetaMorph. Flux is the summation of distances traveled by all moving KIF1A puncta standardized by the length of axon imaged and duration of each movie (in microminutes): $\frac{\sum_{i=1}^{n}d_{i}}{l\times t}$ where d are the individual KIF1A run lengths, l is the length of axon imaged and t is the duration of the imaging session. A KIF1A punctum was defined as undergoing a directed run if it traveled a distance of ≥2 μm. This distance was determined as a safe estimate of the limit of diffusion based on the assumption that root-mean-squared displacement equals $\sqrt{2Dt}$, where *D* is the diffusion coefficient (*D* = 0.01 μs^2^/s for the KIF1A cargo of DCVs) and *t* is the duration of the imaging period (*t* = 50 s; Abney et al., 1999; Gan and Silverman, 2016). A run was defined as terminating if the vesicle remained in the same position for at least four consecutive frames. Percentage flux represents the flux in treated neurons normalized to controls (100%). Statistical analyses were performed using Microsoft Excel or GraphPad Prism. Data are presented as mean ± SEM. Significant differences between treatments were analyzed by *t* tests with equal or unequal variance at a 95% confidence interval. For live imaging experiments, a minimum of 15 cells from three independent cultures (*n* = 3) were analyzed.

### KIF1A immunoprecipitation and GSK3β immunoblotting

Primary hippocampal neurons from E16 WT C57Bl/6 mice (The Jackson Laboratory) were lysed in ice-cold radioimmunoprecipitation assay (RIPA) buffer (50 mm Tris; pH 7.5, 5 mm EDTA, 150 mm NaCl, 1% Triton X-100) containing cOmplete protease inhibitor cocktail (Roche), Halt phosphatase inhibitor cocktail (ThermoFisher), 1 mm phenylmethylsulfonyl fluoride (PMSF), and 1 mm orthovandate. A total of 500 μg of lysate was mixed overnight at 4°C with 12 μg of KIF1A antibody (BD Transduction Laboratories; catalog #612094). Samples were then combined with 50 μl of Protein A/G-agarose (Santa Cruz Biotechnology; catalog #sc-2003) beads and mixed at 4°C for 3 h. Samples were gently pelleted and rinsed three times with RIPA buffer. The immunoprecipitated proteins (5–10 μg) were resolved on 10% SDS-PAGE gels and transferred to polyvinylidene difluoride (PVDF) membranes. Membranes were probed with anti-KIF1A (1:1000, BD Transduction Laboratories; catalog #612094) and anti-GSK3β (1:000, Cell Signaling Technology; catalog #27C10) overnight at 4°C. Immunoreactive bands were visualized using enhanced chemiluminescent substrate (ThermoFisher Scientific) for detection of peroxidase activity from horseradish peroxidase (HRP)-conjugated antibodies.

To detect total KIF1A phosphorylation, C57Bl/6 adult mouse hippocampi (six) were homogenized and lysed in ice-cold RIPA buffer containing cOmplete protease inhibitor cocktail (Roche), Halt phosphatase inhibitor cocktail (ThermoFisher), 1 mm PMSF, and 1 mm orthovandate. 500 μg of lysate was mixed overnight at 4°C with 12 μg of KIF1A antibody (BD Transduction Laboratories; catalog #612094). Samples were then combined with 50 μl of Protein A/G-agarose (Santa Cruz Biotechnology; catalog #sc-2003) beads and mixed at 4°C for 3 h. Samples were gently pelleted and rinsed three times with RIPA buffer. The immunoprecipitated proteins (5–10 μg) were resolved on 10% SDS-PAGE gels and transferred to PVDF membranes. Membranes were probed with an anti-phosphoserine monoclonal antibody 7F12 (1:2000, Invitrogen) and an anti-phosphothreonine monoclonal antibody PT-5H5 (1:2000, Invitrogen) overnight at 4°C. Immunoreactive bands were visualized using enhanced chemiluminescent substrate (SuperSignal West Pico PLUS Chemiluminescent Substrate, ThermoFisher Scientific) for detection of peroxidase activity from HRP-conjugated secondary antibodies.

To determine the presence of KIF1A in COS7 cells, total protein isolated from cell lysates (15 μg) were separated on a precast 4–15% gradient gel (Bio-Rad Laboratories) and subsequently transferred to a PVDF membrane. Membranes were blocked using a 3% BSA solution in TBS-Tween and blotting was performed using primary antibodies in blocking solution against KIF1A (1:500; BD Transduction Laboratories) and the loading control, GAPDH (1:1000; Novus Biologicals; catalog #NB300-327). Immunoreactive bands were visualized using enhanced chemiluminescent substrate (SuperSignal West Pico PLUS Chemiluminescent Substrate: ThermoFisher Scientific) for detection of peroxidase activity from HRP-conjugated secondary antibodies. Blotting was performed in triplicate using two different cell lysate samples.

### Tandem MS (MS/MS) and KIF1A phosphosite analysis

KIF1A was immunoprecipitated from 14-month-old Tg2576 AD (APPSwe) and age-matched WT control mouse brains that were provided by T. Tomiyama (Osaka City University, Japan). Cortices from three brains of each genotype were first homogenized in standard RIPA buffer with 1% NP-40 including cOmplete protease inhibitor cocktail (Roche), Halt phosphatase inhibitor cocktail (ThermoFisher), 1 mm PMSF, and 1 mm orthovandate in a Retsch Mixer Mill 301. Lysates were cleared of insoluble material by centrifugation at 14,000 × *g* for 10 min at 4°C and then incubated with 3.5 μg of anti-KIF1A (BD Transduction Laboratories; catalog #612094) for 3 h at 4°C using constant rotation. Subsequently, 40 μl of Protein A/G PLUS–agarose beads (Santa Cruz Biotechnology; catalog #sc-2003) were added and incubated for another 3 h at 4°C using constant rotation. Immune complexes were washed with 1× RIPA with 0.5 m NaCl and pelleted, followed by a second wash using 2× RIPA with 140 mm NaCl. A total of 5 μg of each sample was heated to 95°C in 2× Laemmli buffer containing 100 mm DTT and resolved on a 10% SDS-PAGE gel. Coomassie-stained protein bands were excised from the SDS-PAGE gel, digested with trypsin, and used for MS/MS with TiO_2_ enrichment for phosphopeptides (University of Victoria Genome BC Proteomics Centre). MS was performed twice on each sample. KIF1A phosphorylation from Tg2576 and WT brain were compared using the algorithm PhosphoRS, which calculates the probability of each phosphorylation site within a peptide. GSK3β phosphosites were identified by comparison to existing sequences within the Phosida (phosida.com) and Phosphonet (https://kinexus.ca) databases.

### Details of MS protocol

#### In gel trypsin digestion

Gel slices were manually cut into 1-mm cubes and Coomassie Blue gel cubes de-stained (50/45/5 v/v methanol/water/acetic acid). Gel cubes were washed with water and 200 mm ammonium bicarbonate before reduction (10 mm dithiothreitol, Sigma) 30 min at room temperature and alkylation (100 mm iodoacetamide, Sigma) 30 min at room temperature. Modified sequencing grade porcine trypsin solution 30 μl (20 ng/μl, Promega) was added to the gel slice enzyme/protein ratio 1:50 and then digested for 16 h at 37°C. The peptides were extracted out of the gel slices with 400 μl (60/39/1 v/v acetonitrile/water/trifluoracetic acid). The sample was then speed vac centrifuged and stored at −80°C until analysis.

#### Titanium dioxide phosphopeptide enrichment

TiO_2_ binding buffer [70% v/v acetonitrile, 5% v/v trifluoroacetic acid (TFA), containing 300 mg/ml lactic acid] was added to 80% of each sample digest along with 10 mg of TiO_2_ beads (GL Science, 10 μm in diameter). Samples were incubated with TiO_2_ with end-over-end rotation for 90 min at 25°C. After incubation, TiO_2_ beads were transferred to homemade StageTip with C_8_ frit for phosphopeptide elution. The beads were washed 4 × 50 μl TiO_2_ binding buffer, and 4 × 50 μl buffer B (80% ACN, 0.1% TFA). Elution was performed with the following steps: 50 μl 0.5% NH_4_OH, 50 μl 0.5% NH_4_OH/30% ACN, and 50 μl 0.5% NH_4_OH/50% ACN. The eluent from the three elutions was pooled and formic acid (20 μl) was added to lower the pH. The samples were frozen at −80°C and lyophilized to dryness before liquid chromatography (LC)-MS analysis.

#### LC-MS/MS analysis

The peptide mixtures were separated by on-line reversed phase chromatography using a Thermo Scientific EASY-nLC II system with a reversed-phase pre-column Magic C-18AQ (100-μm I.D., 2-cm length, 5 μm, 100 Å, Michrom BioResources Inc) pre-column and a reversed phase nano-analytical column Magic C-18AQ (75-μm I.D., 15-cm length, 5 μm, 100 Å, Michrom BioResources Inc) both in-house prepared, at a flow rate of 300 nl/min. The chromatography system was coupled to an LTQ Orbitrap Velos mass spectrometer equipped with a Nanospray II source (ThermoFisher Scientific). Solvents were A: 2% acetonitrile, 0.1% formic acid; B: 90% acetonitrile, 0.1% formic acid. After a 249 bar (∼5 μl) pre-column equilibration and 249 bar (∼8 μl) nanocolumn equilibration, samples were separated by a 55-min gradient (0 min: 5%B; 45 min: 45%B; 2 min: 80%B; 8 min: 80%B). The LTQ Orbitrap Velos (ThermoFisher Scientific) parameters were as follows: nano-electrospray ion source with spray voltage 2.2 kV, capillary temperature 225°C. Survey MS1 scan *m/z* range 400–2000 profile mode, resolution 60,000 at 400 m/z with AGC target 1E6, and one microscan with maximum inject time 200 ms. Lock mass Siloxane 445.120024 for internal calibration with preview mode for FTMS master scans: on, injection waveforms: on, monoisotopic precursor selection: on; rejection of charge state: 1. The eight most intense ions charge state 2–4 exceeding 5000 counts were selected for CID ion trap MSMS fragmentation (ITMS scans 2–9) and detection in centroid mode. Dynamic exclusion settings were: repeat count: 2; repeat duration: 15 s; exclusion list size: 500; exclusion duration: 60 s with a 10 ppm mass window. The CID activation isolation window was: 2 Da; AGC target: 1E4; maximum inject time: 25 ms; activation time: 10 ms; activation Q: 0.250; and normalized collision energy 35%.

#### Data analysis parameters

Raw files were analyzed with Proteome Discoverer 1.3.0.339 software suite (Thermo Scientific). Parameters for the Spectrum Selection to generate peak lists of the CID spectra (activation type: CID; s/n cutoff: 1.5; total intensity threshold: 0; minimum peak count: 1; precursor mass: 350–5000 Da) The peak lists were submitted to an in-house Mascot 2.2 against the Uniprot-Swissprot 20110104 (523,151 sequences; 184,678,199 residues) Allspecies taxonomy and Mouse only database as follows: precursor tolerance 8 ppm; MS/MS tolerance 0.6 Da; Trypsin enzyme 1 missed cleavages; FT-ICR ESI instrument type; fixed modification: carbamidomethylation (C); variable modifications: deamidation (N, Q); oxidation (M), propionamide (C), and phosphorylated (T, Y, S). Percolator settings: max δ Cn 0.05; target FDR strict 0.01, target FDR relaxed 0.05 with validation based on *q* value.

Additional searches were performed against IPI_mouse 3_47 (110 771 sequences; 49,890,456 residues).

### *In vitro* phosphorylation assay

An *in vitro* phosphorylation assay was designed to assess the role of phosphorylation on S402. In this radiometric assay, three different proline directed kinases, cyclin-dependent kinase 2 (CDK2), extracellular signal-regulated kinase 2 (ERK2), and GSK3β, were tested for their ability to phosphorylate the S402 residue on KIF1A by the Kinexus substrate profiling services (Vancouver, BC). The various recombinant protein kinases employed in the substrate profiling process were cloned, expressed and purified using proprietary methods. Quality control testing is routinely performed on each of the kinases to ensure compliance to acceptable standards. The [γ-^33^P] ATP was purchased from PerkinElmer. All other materials were of standard laboratory grade. A total of 2 mg of each peptide was synthesized based on a peptide sequence of interest as listed below:

1. KSP04-CAF KIF1A [S402] WT KKALVGMSPSS-pS-L >98%
2. KSP04-CBT KIF1A [A402] mutant (MT) KKALVGMAPSS-pS-L >98%.

### Inducible Golgi dispersion assay

The rat KIF1A(FL)-V483N coding sequence (Soppina et al., 2014) was tagged with monomeric NeonGreen and an FRB domain. Mutations of S411E and S411A were introduced by QuikChange mutagenesis. Plasmids for expression of WT or mutant motors were cotransfected into COS-7 cells with a plasmid for expression of GMAP210-mRFP-2xFKBP at a ratio of 6:1 with TransIT-LT1 transfection reagent (Mirus). Sixteen hours after transfection, rapamycin (Calbiochem, Millipore Sigma) was added to a final concentration of 44 nm to promote FRB and FKBP heterodimerization and recruitment of motors to the Golgi. After 30 min, the cells were fixed with 3.7% formaldehyde (ThermoFisher Scientific) in 1× PBS for 10 min, quenched in 50 mm ammonium chloride in PBS for 5 min, permeabilized with 0.2% Triton X-100 in PBS for 10 min, and then incubated in blocking buffer (0.2% fish skin gelatin in PBS). Primary [polyclonal antibody against *cis*-Golgi marker giantin (1:1200 PRB-114C, Covance)] and secondary antibodies [Alexa Fluor 680-labeled goat anti-rabbit (1:500, Jackson ImmunoResearch) and DAPI (final concentration 10.9 μm)] were added to blocking buffer and incubated for 1 h each at room temperature. Coverslips were mounted in ProlongGold (Invitrogen) and imaged using an inverted epifluorescence microscope (Nikon TE2000E) with a 40 × 0.75 NA objective and a CoolSnapHQ camera (Photometrics).

Golgi localization before and after motor recruitment was quantified using a distance-based analysis using a custom ImageJ plugin (Budaitis et al., 2019) for *N* ≥ 26 cells across two independent experiments. Briefly, (1) a custom ImageJ plugin generates a line scan from the centroid of the nucleus to the periphery of the cell; this is repeated every one degree for a total of 360 linescans around the cell. The fluorescence intensity along each line scan is determined. (2) For background subtraction, a line scan starting from the centroid of the nucleus to the cell periphery is generated in a region of the cell that lacks cargo and is subtracted from each line scan (scaled background subtraction). Distances that correspond to regions inside the nucleus are removed from each line scan, such that point 0 corresponds to the edge of the nuclear membrane. Oversampling of pixels in the center of the cell was corrected, following the assumption that the cell is a perfect circle. (3) The total distance of each line scan was normalized to itself, such that the distance of each line scan was between 0 (nuclear membrane) and 1 (cell periphery). (4) Pixel intensities were grouped in bins by distance (width, 0.05) and only the top 200 pixels within each bin were included in further analysis. (5) Pixel intensity was averaged for each binned distance to generate a dispersion profile for the cell.

### Statistical analyses

Statistical analyses were performed using Microsoft Excel or GraphPad Prism. For KIF1A live imaging experiments in hippocampal neurons, motor protein flux is presented as mean ± SEM. All experiments were performed on at least 15 cells from three independent cultures. All statistical analyses were performed using one-way ANOVAs with Tukey’s *post hoc* tests, comparing control and treated conditions within the same experiments. For inducible Golgi dispersion assays, the fluorescence intensity for each normalized distance bin was averaged across all cells in two independent experiments and the data were plotted as mean ± SEM. The statistical differences in mean fluorescence intensity were calculated at each binned distance comparing the WT and MT motor for each condition (–Rap and +Rap) using a two-tailed unpaired Student’s *t* test. Differences between the WT and MT motors at *p *<* *0.05 are indicated by an asterisk. WT –Rap, *n* = 26 cells; WT + Rap, *n* = 31 cells; S411A – Rap, *n* = 30 cells; S411A + Rap, *n* = 29 cells; S411E – Rap, *n* = 27 cells; S411E + Rap, *n* = 28 cells.

## Results

### AβOs impair KIF1A transport independent of tau

**Figure 1.**
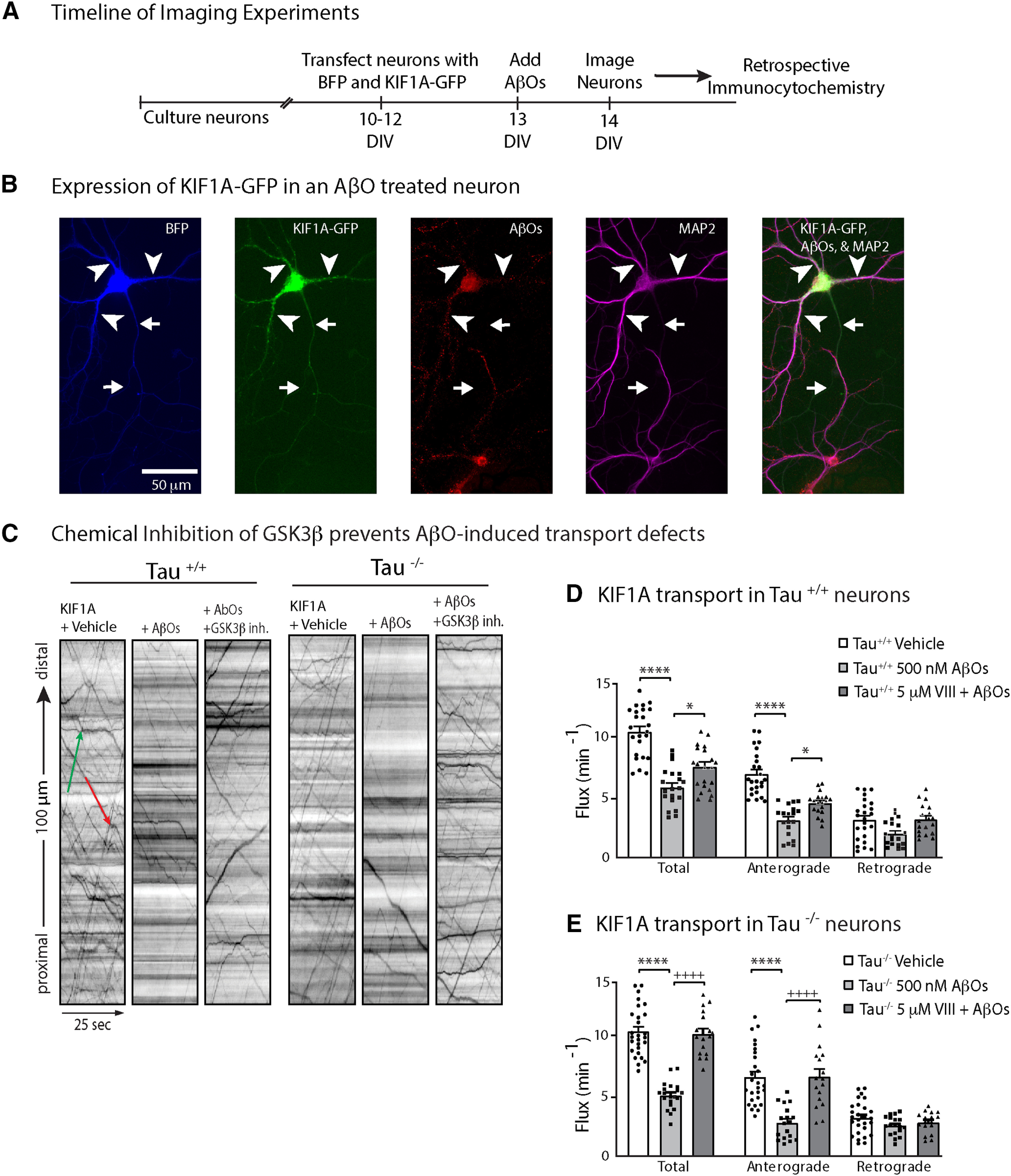
AβO treatment blocks KIF1A transport in a GSK3β-dependent and tau-independent manner. ***A***, Schematic of the timeline of experiments. ***B***, Representative image of soluble BFP and KIF1A-eGFP expressed in an AβO-treated tau^+/+^ neuron. AβOs binds exclusively to dendrites and remain oligomeric after 18 h in culture. Arrows indicate axon; arrowheads indicate dendrites. ***C***, Effects of AβOs and Inhibitor VIII treatment on KIF1A-GFP flux in tau^+/+^ and tau^−/−^ neurons. Representative kymographs comparing KIF1A-GFP transport in control and treated neurons. Lines with a positive slope represent anterograde transport (green); lines with a negative slope represent retrograde transport (red). ***D***, ***E***, Vesicle flux is significantly reduced in tau^+/+^ and tau^−/−^ AβO-treated neurons and is restored by treatment with GSK3β Inhibitor VIII. All bar graphs display mean ± SEM; at least 15 cells from three independent cultures were analyzed. Statistical significance was evaluated by one-way ANOVA with Tukey’s *post hoc* comparisons; *p < 0.05 and ****p < 0.0001 when compared with vehicle; +*p* < 0.05 and ++++*p* < 0.0001 when compared with AβOs; nonsignificant relations are not indicated. For complete transport statistics, see Table 1 and Extended Data Table 1-1.

Previously, Ramser et al. (2013) discovered that AβOs disrupt BDNF transport independent of tau hyperphosphorylation, microtubule destabilization, and neuronal toxicity. These findings raised the intriguing possibility that KIF1A, the primary kinesin motor required for BDNF transport (Barkus et al., 2008; Lo et al., 2011; Kondo et al., 2012), is directly impaired in AβO-treated neurons by a mechanism independent of tau. In the present study, we cultured hippocampal neurons from WT (tau^+/+^) and tau knock-out (tau^−/−^) mice, expressed mouse KIF1A tagged with enhanced green fluorescent protein (KIF1A-eGFP) in these neurons at 10 DIV, and added vehicle solution (control) or 500 nm AβOs to their culture medium at 11 DIV (Fig. 1*A*). Irreversible AβO binding was confirmed retrospectively by immunocytochemistry (Fig. 1*B*) using an oligomer-selective antibody (NU-4; Lambert et al., 1998). After 18 h of treatment, we imaged KIF1A-eGFP transport and compared the flux, velocity, and run length of axonal KIF1A puncta across all conditions (Fig. 1*C*; Table 1). Representative kymographs illustrate differences between KIF1A transport in control and AβO-treated neurons (Fig. 1*C*). AβO treatment dramatically reduced anterograde KIF1A flux in both tau^+/+^ and tau^−/−^ neurons, indicating that tau is not required for AβO disruption of KIF1A transport (Fig. 1*D*,*E*; Table 1). AβOs also significantly decreased anterograde average velocity and run length in tau^−/−^ neurons. Retrograde movement of KIF1A, presumably driven by cytoplasmic dynein, was also decreased on AβO treatment, although the effect in tau^−/−^ neurons was not statistically significant (Table 1). In all experiments, KIF1A transport parameters were similar to those previously reported for BDNF (Lo et al., 2011; Seifert et al., 2016; Bodakuntla et al., 2020), implying that changes in KIF1A motility are not attributed to overexpression artefacts. These results demonstrate that AβOs reduce KIF1A motility independent of tau.

**Table 1:** Quantitative analysis of axonal KIF1A-GFP transport in tau**^+/+^** **and tau^−/−^** **neurons**.

	Traffic values		
	All Events	Anterograde	Retrograde
Flux (min–1)			
tau^+/+^ vehicle	10.46 ± 0.48	7.03 ± 0.78	3.35 ± 0.38
tau^+/+^ AβOs	5.83 ± 0.24^****^ [Fn TF10]	2.71 ± 0.65^****^	3.12 ± 0.35
tau^+/+^ VII + AβOs	7.56 ± 0.53^*#^	4.19 ± 0.35^#^	3.37 ± 0.40
tau^−/−^ vehicle	10.74 ± 0.70	7.38 ± 0.35	3.35 ± 0.33
tau^−/−^ AβOs	5.77 ± 0.43^****^	3.11 ± 0.25^****^	2.66 ± 0.32
tau^−/−^ VII + AβOs	10.38 ± 1.01^####^	7.42 ± 0.76^####^	2.95 ± 0.38
Velocity (μm/s)			
tau^+/+^ vehicle	1.68 ± 0.12	2.04 ± 0.16	1.33 ± 0.12
tau^+/+^ AβOs	1.63 ± 0.12	1.72 ± 0.17	1.31 ± 0.16
tau^+/+^ VII + AβOs	1.75 ± 0.08	1.48 ± 0.18	1.47 ± 0.09
tau^−/−^ vehicle	1.93 ± 0.07	2.28 ± 0.14	1.55 ± 0.07
tau^−/−^ AβOs	1.41 ± 0.18^*^	1.54 ± 0.19^*^	1.30 ± 0.19
tau^−/−^ VII + AβOs	1.98 ± 0.11^#^	2.35 ± 0.16^#^	1.54 ± 0.12
Run length (μm)			
tau^+/+^ vehicle	18.85 ± 1.09	26.02 ± 2.02	12.09 ± 1.30
tau^+/+^ AβOs	17.26 ± 1.65	19.74 ± 2.83	13.37 ± 2.00
tau^+/+^ VII + AβOs	17.48 ± 0.94	23.05 ± 1.67	10.96 ± 0.76
tau^−/−^ vehicle	20.39 ± 1.05	27.32 ± 2.13	13.75 ± 1.21
tau^−/−^ AβOs	11.27 ± 0.88^****^	13.38 ± 1.11^***^	9.82 ± 0.91
tau^−/−^ VII + AβOs	20.79 ± 2.01^####^	29.38 ± 3.77^###^	12.64 ± 0.72

tau^+/+^ vehicle: *n* = 24 kymographs (24 cells, 893 puncta).

tau^−/−^ vehicle: *n* = 27 kymographs (27 cells, 1014 puncta).

tau^+/+^ AβO: *n* = 18 kymographs (19 cells, 432 puncta).

tau^−/−^ AβO: *n* = 18 kymographs (18 cells, 578 puncta).

tau^+/+^ VIII + AβO: *n* = 18 kymographs (18 cells, 643 puncta).

tau^−/−^ VIII + AβO: *n* = 17 kymographs (17 cells, 638 puncta).

^*^p < 0.05, when compared with vehicle.

^**^p < 0.01, when compared with vehicle.

^***^p < 0.001, when compared with vehicle.

^****^p < 0.0001, when compared with vehicle.

^#^*p* < 0.05, when compared with AβOs.

^##^*p* < 0.01, when compared with AβOs.

^###^*p* < 0.001, when compared with AβOs.

^####^*p* < 0.0001, when compared with AβOs.

10.1523/ENEURO.0176-20.2020.t1-1 Extended Data Table 1-1 ANOVA outputs for trafficking values. Download Table 1-1, DOCX file.

### GSK3β inhibition prevents KIF1A transport defects in AβO-treated neurons

Pharmacological inhibition and dominant negative mutations of GSK3β prevent BDNF transport defects induced by AβOs (Ramser et al., 2013). This effect is observed in both tau^+/+^ and tau^−/−^ neurons, ruling out the possibility that tau activates PP1-GSK3β signaling to block transport, as reported previously for KIF5 in isolated squid axoplasm (Kanaan et al., 2011). Does GSK3β impair transport by reducing KIF1A motility in AβO-treated neurons, and does GSK3β require tau for this function? We incubated tau^+/+^ and tau^−/−^ neurons with 5 μm Inhibitor VIII (a selective, cell-permeable, competitive blocker of GSK3β; Bhat et al., 2003) for 30 min before AβO treatment. Pretreatment with Inhibitor VIII prevented defects in KIF1A flux, velocity and run length (Fig. 1*C–E*; Table 1). Thus, consistent with previous findings for BDNF transport (Ramser et al., 2013), GSK3β mediates KIF1A transport impairment in AβO-treated neurons. This observed effect was comparable in both tau^+/+^ and tau^−/−^ neurons (Fig. 1*B*; Table 1), demonstrating that GSK3β reduces KIF1A motility independent of tau.

### KIF1A interacts with GSK3β and is phosphorylated at a conserved GSK3β consensus site

**Figure 2.**
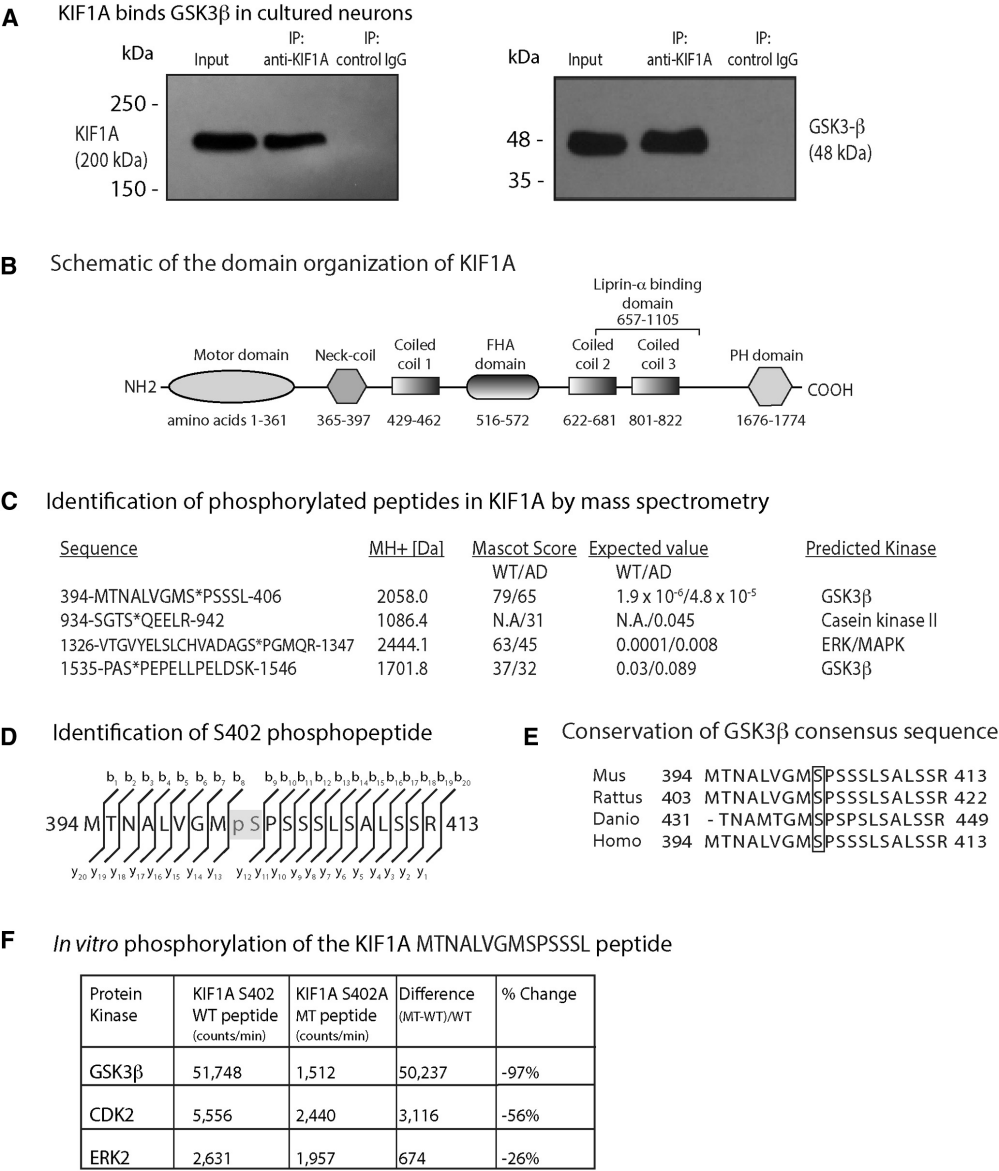
GSK3β phosphorylates KIF1A at several sites. ***A***, A monoclonal antibody to KIF1A immunoprecipitated GSK3β from neuronal lysates. ***B***, Schematic of the domain organization of KIF1A (GenBank BAA0622.1). ***C***, Tandem mass spectroscopy on KIF1A isolated from WT and AD model mouse (APPswe; AD) brain identified four phosphopeptides which conform to sites targeted by kinases that are aberrantly activated in AD, such as MAPK, CK2, and GSK3β. Serine 402 conforms to a GSK3β phosphorylation site according to Phosida (http://141.61.102.18/phosida/index.aspx) and Phosphonet (https://kinexus.ca). ***D***, MS identification of the *b+* and *y+* ions that are the direct (N to C terminus) and reverse (C to N terminus) ion series obtained during collision-induced dissociation (also see Extended Data Fig. 1-1). ***E***, Sequence comparison of the region surrounding Serine 402 between zebrafish, mouse, rat, and human proteins. ***F***, The evaluation of three different proline-directed kinases (CDK2, ERK2, and GSK3β) by radiometric assay indicates that GSK3β shows a strong preference for the KIF1A (S402) WT peptide over the A402 MT peptide *in vitro*.

GSK3β has been shown to associate with kinesin-1 motor complexes (Morfini et al., 2002). To determine whether KIF1A interacts with GSK3β, we generated cell lysates from mouse hippocampi and immunoprecipitated KIF1A using a specific monoclonal antibody (Fig. 2*A*). By immunoblotting, we found that GSK3β coimmunoprecipitates with input and KIF1A proteins, but not with negative control IgG proteins alone (Fig. 2*A*). This result indicates that KIF1A and GSK3β interact, either by forming a direct complex or by binding indirectly through scaffold proteins.

To identify specific residues of KIF1A that are phosphorylated by GSK3β, we isolated KIF1A from 14-month-old WT and Tg2576 (APP_Swe_) mouse brains by immunoprecipitation and resolved the proteins by SDS-PAGE. Following in-gel tryptic digestion, the resulting peptides were analyzed by sequential LC-MS/MS. Although immunoblots with pan-phospho-Ser and pan-phospho-Thr antibodies failed to detect any phospho-KIF1A immunoreactivity (Extended Data Fig. 1-1), we detected nine phosphopeptides in KIF1A (UniProtKB/Swiss-Prot: Q6TA13), four of which conform to sequences targeted by kinases that are aberrantly activated in AD including mitogen-activated protein kinase (MAPK), casein kinase II (CK2), and GSK3β (Fig. 2*B*,*C*; Extended Data Fig. 2-1; Maqbool et al., 2016; Cline et al., 2018). Because our analyses were not quantitative, we were unable to establish whether these phosphosites were more abundant in Tg2576 mice relative to WT mice. We compared high confidence KIF1A phosphopeptides in WT and Tg2576 mice using PhosphoRS and mapped them to specific sites within KIF1A functional domains (Fig. 2*B*). Phosphorylation within specific KIF1A sequences varied between genotypes; for example, S937, located within the presumed cargo-binding peptide SGTS*QEELR, was phosphorylated (denoted by *) in Tg2576 mice but not detected in WT mice (Fig. 2*D*). However, S937 did not conform to an AD-related kinase motif according to the Phosida and Phosphonet databases. Based on these analyses, we focused on S402 (Fig. 2*D*) as it (1) is located between the neck-coil domain and the first coiled-coil domain and could therefore impact motor dimerization and/or motility (Fig. 2*B*; Al-Bassam et al., 2003; Hammond et al., 2009; Soppina et al., 2014), (2) conforms to a GSK3β consensus site, and (3) is conserved between zebrafish, mouse, rat, and human KIF1A proteins (Fig. 2*E*).

10.1523/ENEURO.0176-20.2020.f1-1 Extended Data Figure 1-1 Pan-serine and pan-threonine antibodies fail to detect phosphorylated KIF1A by immunoblotting in adult mouse hippocampi. Download Figure 1-1, TIF file.

10.1523/ENEURO.0176-20.2020.f2-1 Extended Data Figure 2-1 Mass spectrum of the KIF1A MTMALVGNS*PSSSLSALSSR phosphopeptide. ***A***, The graph shows the output mass spectrum, obtained from the mass spectrometer, for one of the identified phosphopeptides of the KIF1A protein. The graph plots ion intensity versus mass to charge ion ratio (M/Z) for *b+* (red) and *y+* (blue) ions that are the direct (N to C terminus) and reverse (C to N terminus) ion series obtained during collision-induced dissociation (CID). Also see Figure 2 in the main text. The detected ions of the b-ion and y-ion collision series are shown in the inset; *denotes phosphorylated residue. ***B***, Identification of other phosphopeptides of KIF1A. No known kinase motifs were identified in these sequences. Download Figure 2-1, TIF file.

### GSK3β phosphorylates KIF1A at S402 *in vitro*

To confirm phosphorylation of S402 and evaluate whether GSK3β can perform this modification, we developed a sensitive *in vitro* radiometric enzyme assay (Fig. 2*F*). We synthesized a WT KIF1A peptide containing S402 and a phosphomutant KIF1A peptide containing an alanine substitution at S402 (S402A). We then tested three different proline-directed kinases (CDK2, ERK2, and GSK3β) for their ability to phosphorylate these peptides. Mutation of S402 nearly abolished the ability of GSK3β to phosphorylate the S402-containing peptide whereas the kinase activities of CDK2 and ERK2 were less impacted by the S402A mutation (Fig. 2*F*). These results confirm that S402 is phosphorylated by GSK3β *in vitro* and support a plausible role for this residue in regulating KIF1A motility.

### Point mutations of S402 do not alter KIF1A motility

**Figure 3.**
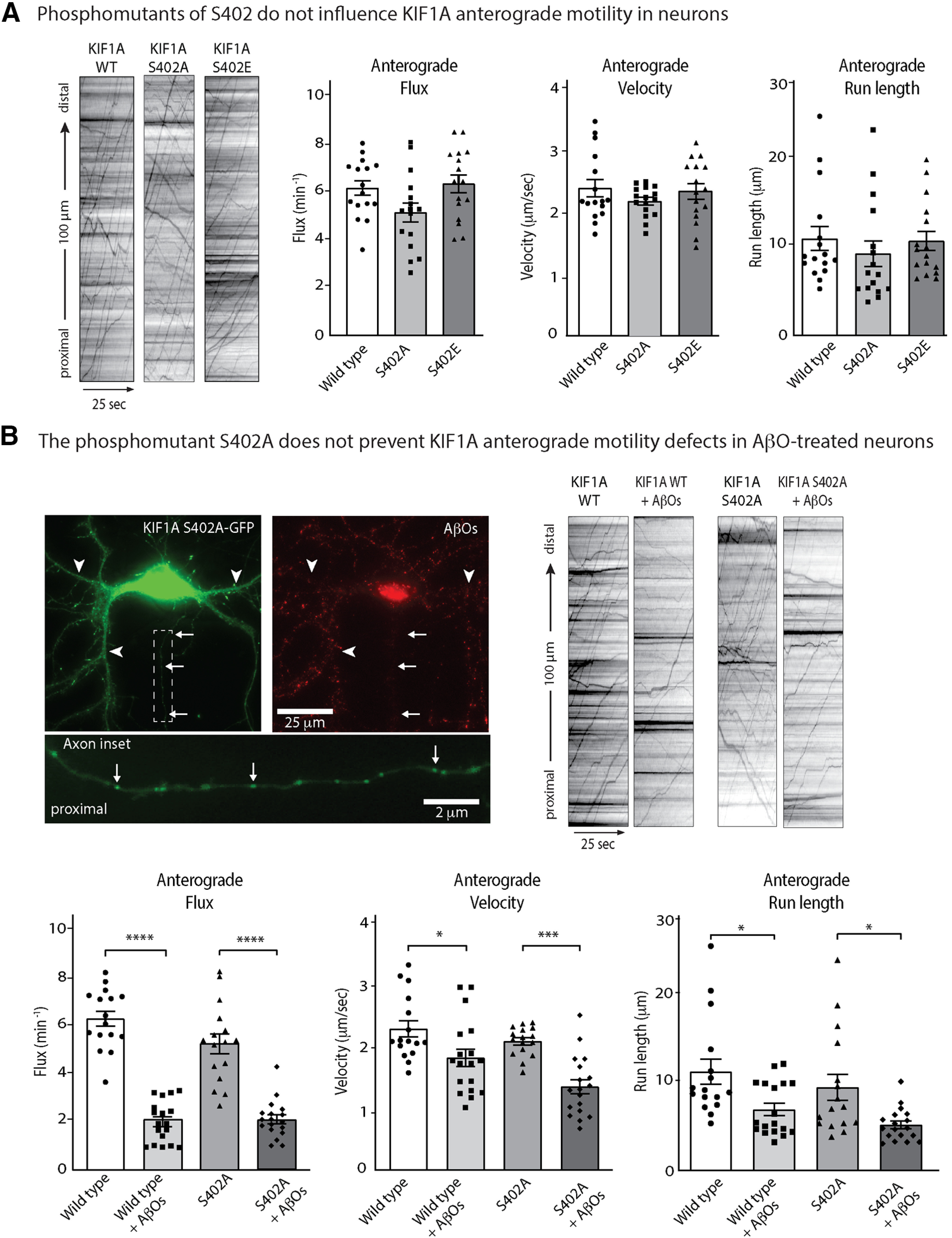
Phospho-mutants of Serine402 do not alter KIF1A-mediated transport. ***A***, Representative kymographs comparing KIF1A-eGFP WT, S402A, and S402E transport in WT neurons. Bar graphs display mean ± SEM; at least 15 cells from three independent cultures were analyzed. Statistical significance was evaluated by one-way ANOVA with Tukey’s *post hoc* comparisons; nonsignificant relations are not indicated. For complete statistics, see Table 2 and Extended Data Table 1-1. ***B***, Representative image of KIF1A S402A-eGFP expressed in an AβO-treated WT neuron. AβOs bind exclusively to dendrites and remain oligomeric after 18 h in culture. Arrows indicate axon; arrowheads indicate dendrites. Representative kymographs comparing KIF1A-eGFP WT and S402A transport in control and AβO-treated neurons. Bar graphs display mean ± SEM; at least 15 cells from three independent cultures were analyzed. Statistical significance (*p <* *0.05, **p <* *0.01, *** *p *<* *0.001, ****p <* *0.0001) was evaluated by one-way ANOVA with Tukey’s *post hoc* comparisons; nonsignificant relations are not indicated. For complete statistics, see Table 2 and Extended Data Table 1-1.

Does phosphorylation at S402 regulate KIF1A motility? We generated a non-phosphorylatable form of KIF1A-eGFP by inducing a Ser-to-Ala point mutation (KIF1A-S402A) at this site. Conversely, we induced a Ser-to-Glu point mutation to mimic GSK3β phosphorylation of KIF1A-eGFP at this site (KIF1A-S402E). We expressed these mutants in mouse hippocampal neurons and compared their motility to WT KIF1A by live cell imaging (Fig. 3*A*). Representative kymographs and quantification of transport parameters revealed no significant differences in flux, run length and velocity for KIF1A-S402A or KIF1A-S402E relative to WT KIF1A (Fig. 3*A*; Table 2; Extended Data Table 1-1). These data imply that S402 phosphorylation does not regulate KIF1A transport in hippocampal neurons. We next asked whether AβO-induced overactivation of GSK3β impairs KIF1A transport via phosphorylation at S402. We compared the motility of WT KIF1A-eGFP and KIF1A-S402A-eGFP in control and AβO-treated neurons (Fig. 3*B*; Table 2; Extended Data Table 1-1). Kymograph analyses revealed that AβO treatment similarly reduced the anterograde fluxes, velocities and run lengths of the WT and mutant motors, indicating that the alanine point mutation does not protect against KIF1A transport defects. Taken together, these results indicate that GSK3β does not regulate KIF1A motility through phosphorylation at S402A.

**Table 2:** Quantitative analysis of axonal KIF1A-S402A and KIF1A-S402E transport in tau**^+/+^ neurons**

	Traffic values		
	All events	Anterograde	Retrograde
Flux (min–1)			
WT	8.00 ± 0.46	6.11 ± 0.30	1.88 ± 0.30
WT + ABO	3.05 ± 0.46^****####^	1.94 ± 0.20^****####^	1.12 ± 0.20^####^
S402A	7.72 ± 0.34	5.10 ± 0.40	2.71 ± 0.27
S402A + ABO	3.80 ± 0.24^****####^	2.04 ± 0.18^****####^	2.71 ± 0.27^#^
S402E	7.63 ± 0.42	6.30 ± 0.37	1.34 ± 0.17^##^
Velocity (μm/s)			
WT	2.05 ± 0.11	2.28 ± 0.13	1.53 ± 0.14
WT + ABO	1.72 ± 0.14	1.81 ± 0.14^*^	1.55 ± 0.15
S402A	2.03 ± 0.07	2.08 ± 0.06	1.96 ± 0.15
S402A + ABO	1.23 ± 0.10^****####$$^	1.35 ± 0.12^****###$^	1.13 ± 0.11^###^
S402E	2.03 ± 0.10	2.23 ± 0.12	1.51 ± 0.12
Run length (μm)			
WT	9.14 ± 1.02	10.74 ± 1.36	5.64 ± 0.57
WT + ABO	6.26 ± 0.62^*^	6.62 ± 0.68^*^	5.64 ± 0.89
S402A	7.34 ± 0.90	9.03 ± 1.42	5.31 ± 0.39
S402A + ABO	4.77 ± 0.29^***^	4.93 ± 0.42^***#^	4.53 ± 0.25
S402E	9.09 ± 0.90	10.46 ± 1.08	5.31 ± 0.54

WT: *n* = 16 kymographs (16 cells, 789 puncta).

S402A: *n* = 16 kymographs (16 cells, 829 puncta).

WT + ABO: *n* = 18 kymographs (18 cells, 381 puncta).

S402A + ABO: *n* = 18 kymographs (18 cells, 430 puncta).

S402E: *n* = 16 kymographs (16 cells, 743 puncta).

^*^p < 0.05, when compared with WT.

^**^p < 0.01, when compared with WT.

^**^^*^p < 0.001, when compared with WT.

^****^p < 0.0001, when compared with WT.

^#^*p* < 0.05, when compared with S402A.

^##^*p* < 0.01, when compared with S402A.

^###^*p* < 0.001, when compared with S402A.

^####^*p* < 0.0001, when compared with S402A.

^$^*p* < 0.05, when comparing AβO-treated conditions (significance denoted in S402 + AβO condition).

^$$^*p* < 0.01, when comparing AβO-treated conditions (significance denoted in S402 + AβO condition).

^$$$^*p* < 0.001, when comparing AβO-treated conditions (significance denoted in S402 + AβO condition).

^$$$$^*p* < 0.0001, when comparing AβO-treated conditions (significance denoted in S402 + AβO condition).

**Figure 4.**
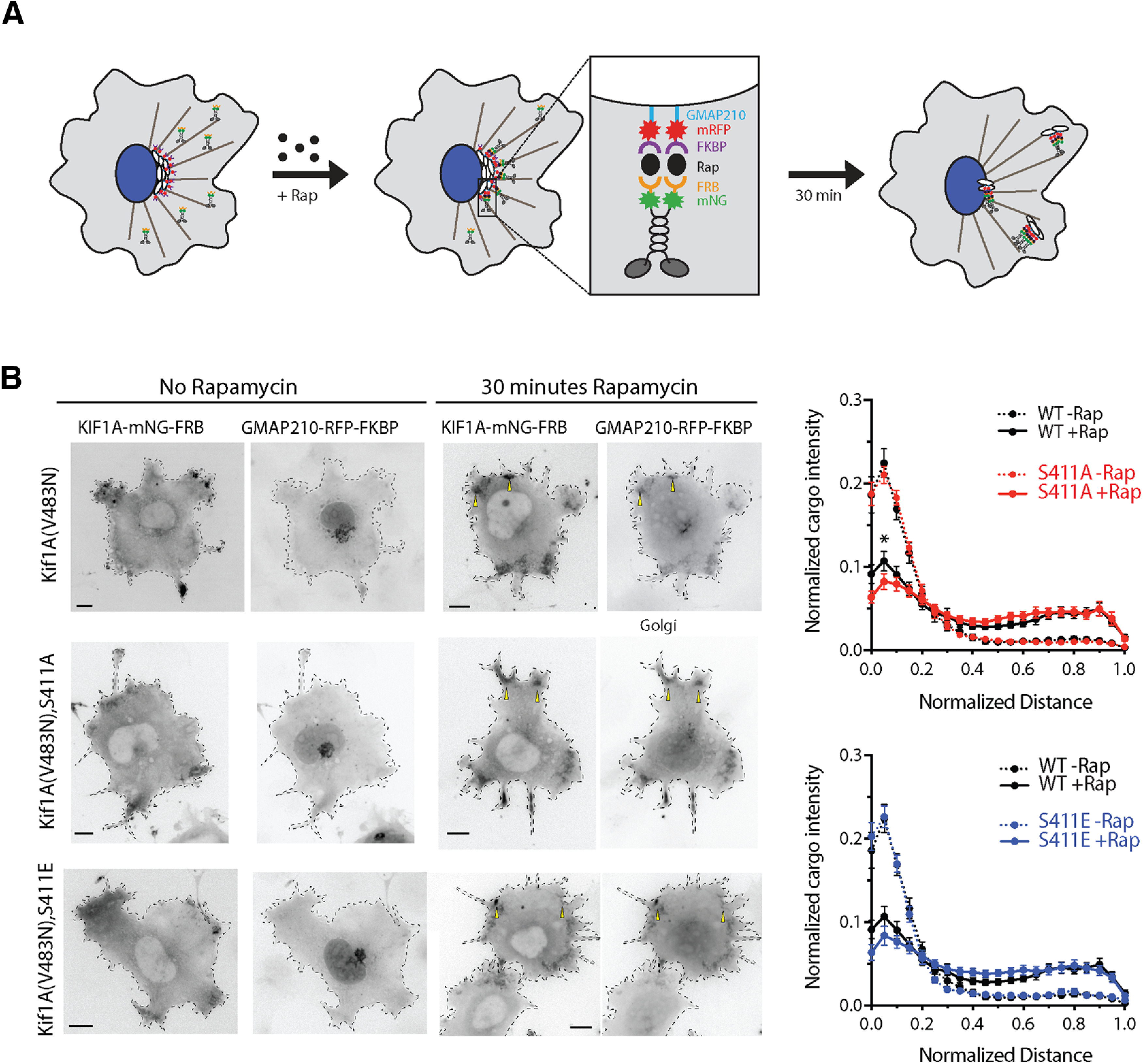
A phospho-mutant of S402 does not influence KIF1A activity in Golgi-dispersion assays. ***A***, Schematic of the inducible motor recruitment assay. A kinesin motor tagged with monomeric NeonGreen (mNG) and an FRB domain (KIF1A-mNG-FRB) is coexpressed with a Golgi targeting sequence (GMAP210) tagged with mRFP and FKBP domain (GMAP210-mRFP-FKBP) in COS7 cells. Addition of rapamycin (+Rap) causes heterodimerization of the FRB and FKBP domains and recruitment of motors to the cargo membrane. Recruitment of active motors drives cargo dispersion to the cell periphery. ***B***, COS7 cells expressing WT, S411A, or S411E versions of full-length active (V483N) KIF1A-mNG-FRB together with GMAP210-mRFP-2xFKBP were treated with ethanol vehicle (no rapamycin, left panels) or with 44 nm rapamycin (middle panels). After 30 min, the cells were fixed and stained with an antibody to the Golgi marker giantin (data not shown) and DAPI (data not shown). Representative images of the KIF1A-mNG-FRB (KIF1A motor) and GMAP210-mRFP-2xFKBP (Golgi marker) channels are shown in inverse grayscale. Yellow arrowheads indicated examples of dispersed Golgi particles. Scale bars: 10 μm. The Golgi distribution in the giantin channel was quantified and the data are displayed as the fluorescence intensity of Golgi particles on the *y*-axis and the normalized distance on the *x*-axis (0.0 = edge of nucleus, 1.0 = plasma membrane). WT –Rap (dotted black lines), *n* = 26 cells; WT +Rap (solid black lines), *n* = 31 cells; S411A –Rap (dotted red line), *n* = 30 cells; S411A +Rap (solid red line), *n* = 29 cells; S411E –Rap (dotted blue line), *n* = 27 cells; S411E +Rap (solid blue line), *n* = 28 cells across two independent experiments. Statistical differences in mean cargo intensity between WT and MT motors were calculated at each binned distance and for each condition (–Rap and +Rap) by using a two-tailed unpaired Student’s *t* test; * *p *<* *0.05. For complete statistical analysis, see Extended Data Figure 4-2.

As an alternative explanation for this outcome, endogenous WT KIF1A may dimerize with mutant KIF1A monomers or preferentially bind to cargo, thus masking any effects of the mutations on KIF1A motility. To investigate the effects of S402A and S402E point mutations on KIF1A motility in the absence of endogenous KIF1A transport, we performed an inducible Golgi dispersion assay in COS7 cells (Engelke et al., 2016; Budaitis et al., 2019; Fig. 4*A*; Extended Data Figs. 4-1, 4-2). In this assay, Golgi-targeted kinesin motors cause the dispersion of the Golgi complex from its characteristic tightly-packed, perinuclear location to a phenotype consisting of small Golgi-derived vesicles scattered throughout the cytoplasm (Engelke et al., 2016). We used the rapamycin-induced dimerization of FRB and FKBP to drive the rapid recruitment of KIF1A motors to the Golgi surface and determined the effect on Golgi dispersion after 30 min of motor activity. This assay probes the ability of motors to drive transport in a cellular environment and requires motors capable of not only processive motility, but also sufficient force generation to oppose the Golgi-localized dynein that is responsible for the perinuclear clustering of this organelle (Corthésy-Theulaz et al., 1992; Burkhardt et al., 1997).

10.1523/ENEURO.0176-20.2020.f4-1 Extended Data Figure 4-1 KIF1A is undetectable in COS7 cells. Immunoblot analysis of endogenous KIF1A expression in neuronal and non-neuronal cells. KIF1A is detectable in primary hippocampal neurons and SK-N-SH lysates. A second band is detected in the SK-N-SH cells which may indicate the presence of a KIF1A isoform, for which at least five have been identified (https://www.ncbi.nlm.nih.gov/genbank/). Download Figure 4-1, TIF file.

10.1523/ENEURO.0176-20.2020.f4-2 Extended Data Figure 4-2 Student’s t test *p* values for Golgi dispersion assay. Download Figure 4-2, DOCX file.

Because full-length KIF1A resides in an inactive, autoinhibited state, we first introduced a point mutation (V483N) that relieves the self-inhibition of motor activity (Huo et al., 2012; Yue et al., 2013; Soppina et al., 2014). Using this active version of full-length KIF1A, we then introduced point mutations that could abolish or mimic the phosphorylation state of the S402 residue. Using the rat KIF1A sequence, these mutations are S411A and S411E, respectively. WT and MT full-length KIF1A(V483N) motors were fused to mNeonGreen and a FRB domain, whereas the Golgi targeting sequence of the *cis*-Golgi resident protein GMAP210 was fused to monomeric red fluorescent protein (mRFP) and the FKBP domain (Engelke et al., 2016). Addition of rapamycin resulted in rapid recruitment of WT, S411A, and S411E motors to the Golgi surface and subsequent transport of Golgi particles to the cell periphery. No differences in Golgi dispersion were observed between the WT, S411A and S411E motors (Fig. 4*A*; Extended Data Fig. 4-2). These results indicate that the phosphorylation state of S411 does not impact the motility and force generation of KIF1A in this system. Alternatively, it is possible that the Ser-to-Glu mutation does not mimic KIF1A phosphorylation in this context (Dephoure et al., 2013).

## Discussion

Impairment of axonal transport is an early pathologic event that leads to axonal degeneration and loss of synapse function in AD (Stokin et al., 2005; Minoshima and Cross, 2008; Gallagher et al., 2012; Sleigh et al., 2019). Soluble AβOs, a causative agent of AD (Cline et al., 2018), activate intracellular signaling cascades that trigger aberrant phosphorylation of many target proteins, including tau. Many studies have examined how tau hyperphosphorylation, microtubule destabilization, and cytoskeletal collapse impair transport (Mandelkow et al., 2003; Stokin and Goldstein, 2006; Cowan et al., 2010; Combs et al., 2019). However, subtler mechanisms of AβO-induced transport disruption that arise before neurotoxicity and that act directly on motor proteins to reduce their motility have received much less attention and have not been investigated in mammalian neurons.

Here, through direct assessment of trafficking at high spatial and temporal resolution in mouse hippocampal neurons, we demonstrate that AβOs impair KIF1A motility independent of tau (Fig. 1). These results are consistent with previous findings that AβOs impair motility of the KIF1A cargo protein BDNF and that this defect is independent of tau (Ramser et al., 2013). We also demonstrate that pharmacological inhibition of GSK3β prevented these transport defects (Fig. 1), motivating us to investigate whether KIF1A motility is dysregulated by phosphorylation. By MS, we identified nine phosphorylation sites on KIF1A, four of which are targeted by kinases associated with AD, including GSK3β (Fig. 2). We discovered that S402, located proximal to the neck-coil domain of KIF1A, conforms to a highly conserved GSK3β consensus site (Fig. 2). Using an *in vitro* kinase assay, we confirmed that S402 is indeed phosphorylated by GSK3β (Fig. 2). Finally, we tested whether a phosphomimic of S402 could modulate KIF1A motility in cultured neurons and in a Golgi dispersion assay using COS-7 cells that lacked endogenous KIF1A. Unfortunately, in both experimental systems, no significant differences in transport properties were observed between WT and mutant KIF1A (Figs. 3 and 4). From these findings, we conclude that GSK3β impairs KIF1A transport independent of tau but likely does not achieve this effect by phosphorylating KIF1A at S402.

Traditionally, axonal transport defects were viewed as a consequence of tau hyperphosphorylation and aggregation, tau-induced kinase activation, microtubule dissolution, and axonal dystrophy during the advanced stages of disease progression (Mandelkow et al., 2003; Stokin and Goldstein, 2006; LaPointe et al., 2009; Morfini et al., 2009; Kanaan et al., 2011). However, transport defects can occur independent of tau and before overt morphologic decline and cell death, suggesting that early transport defects might play a causal role in AD pathogenesis (Pigino et al., 2003; Lazarov et al., 2007; Stokin et al., 2008; Goldstein, 2012; Rodrigues et al., 2012; Ramser et al., 2013). Recent reports indicate that overactivation of GSK3β contributes to many pathologic hallmarks of AD including increased Aβ production, tau hyperphosphorylation, and impaired learning and memory (Maqbool et al., 2016). Data from our present study suggest, pending identification of the specific phosphorylation sites, that the pathologic activation of GSK3β can impair transport by earlier, subtler mechanisms that involve inhibitory phosphorylation of motor proteins. Such mechanisms of GSK3β-dependent motor protein inhibition have been characterized previously in invertebrate models of AD. As a negative regulator of axonal transport in *Drosophila*, active GSK3β binds and phosphorylates kinesin-1 and reduces the number of motors bound to microtubules (Weaver et al., 2013; Banerjee et al., 2018). Alternatively, kinesin motor activity can be inhibited by cargo detachment; in isolated squid axoplasm treated with AβOs, GSK3β phosphorylates kinesin light chain-2, promoting motor-cargo dissociation and impairing transport (Morfini et al., 2002). Interestingly, and perhaps paradoxically, we found a significant effect on KIF1A run lengths and velocity in the tau^−/−^ neurons treated with AβOs (Fig. 1; Table 1). AβOs dysregulate a number of intracellular cascades, including those active toward tau (Cline et al., 2018). It is possible that in the absence of a tau-kinase substrate, these kinases (or phosphatases) aberrantly target regulatory sites on KIF1A that govern processivity akin to the phosphorylation of the motor domain on kinesin-1 in *Drosophila* (Weaver et al., 2013; Banerjee et al., 2018) and KIF5C at S176 (DeBerg et al., 2013). Our study is congruent with those reports and, additionally, is first to show that GSK3β impairs KIF1A motility in a mammalian primary culture model of AD (Fig. 1).

From the nine phosphorylation sites on KIF1A revealed by MS (Fig. 2; Extended Data Fig. 1-1), we chose to investigate S402. This phosphopeptide has also been identified by large scale phosphoproteomic analyses in mouse and human brain (Ullah et al., 2016). However, we found that point mutations of S402 do not regulate KIF1A transport in two cell-based assays (Figs. 3 and 4). As these are negative results, it is possible that the S402E mutation does not fully mimic the addition of a phosphate at this site in the WT motor. It is also possible that single amino acid changes meant to prevent or mimic phosphorylation events are insufficient to alter motor behavior. Indeed, another study found that AMP kinase-dependent phosphorylation of kinesin light chain-1 (KLC1) at S517/S520 does not affect motor function (McDonald et al., 2010). However, several reports demonstrate that increased phosphorylation at a single site is sufficient to impair motor protein motility. Phosphorylation of KIF5C at S176 by JNK weakens motor-microtubule interactions and/or reduces force output (DeBerg et al., 2013; Padzik et al., 2016). Cargo binding, which is required to activate motor proteins, is also disrupted by phosphorylation. MAP kinase phosphorylates KLC1 at S460 to reduce binding and trafficking of calsyntenin-1 (Vagnoni et al., 2011). Moreover, S460 phosphorylation is increased in AD, disrupting axonal transport and promoting amyloidogenic processing of the amyloid precursor protein (APP; Mórotz et al., 2019). Therefore, it may be that phosphorylation at multiple sites synergistically impairs motor motility. Future studies on KIF1A could investigate whether GSK3β and/or other kinases phosphorylate different sites within the motor and cargo-binding domains, and whether mechanisms of cargo dissociation and microtubule detachment converge to impair KIF1A motility.

## Synthesis

Reviewing Editor: Eran Perlson, Tel Aviv University Decisions are customarily a result of the Reviewing Editor and the peer reviewers coming together and discussing their recommendations until a consensus is reached. When revisions are invited, a fact-based synthesis statement explaining their decision and outlining what is needed to prepare a revision will be listed below.

The manuscript “GSK3ß impairs KIF1A transport in a cellular model of Alzheimer’s disease but does not regulate motor motility at S402 “ characterizes the contribution of GSK3β on axonal transport in AD model. The authors demonstrate that AβOs treatment impair axonal transport process via GSK3β pathway. Furthermore, the authors found phosphorylated residues by GSK3β on KIF1 and test specifically if phosphorylated S402 can explain KIF1A transport impairment. Unfortunately, this was not the case. Although negative result, this observation is highly important to the field and should be published.

Still as the authors didn’t demonstrate directly that GSK3β ‘does not regulate motor motility at S402’, and there are several important points needed to be clarified, the manuscript at its current stage is premature for publication.

The authors should address this specific point:

The authors see no difference in axonal transport/Golgi relocalisation when over expressing KIF1A phospho-mutants (Fig. 3). This could be due to the fact that the Ser-to-Glu mutation might not properly mimic KIF1A phosphorylation in these contexts, as they also rightly point out (p. 15, 17). It is also possible, however, that the endogenous levels of phosphorylated S402 are low in WT neurons (have these been used in Fig. 3A?). In this case, over expressing the S402A mutant, which they can be confident cannot be phosphorylated, is likely to have little effect. In absence of quantitative mass spectrometry, authors should challenge the neurons with AβOs (as in Fig. 1) or over express a constitutively active form of GSK3β (predicted to increase S402 phosphorylation and to impair transport) in KIF1A WT and S402A backgrounds. If S402 phosphorylation by GSK3β does not mediate KIF1A transport, then there should be no difference between WT and S402A backgrounds (i.e. the alanine mutant is not protective against transport reduction) and one could rule out that the effect of GSK3β on transport is via S402.

The authors should also clarified these points:

Fig.1/Table 1

The difference between run lengths and fluxes needs to be clarified. In the Materials and Methods the authors define fluxes as total distance traveled by KIF1A puncta (p. 5), i.e. standardised summation of run lengths. Do they mean summation of runs instead? This is important when considering Table 1. From the analysis of KIF1A motility, it seems that the absence of Tau is generally necessary for the AβOs and inhibitor VIII-mediated effect on velocity and run length, but not on fluxes. If the fluxes do reflect the number of moving vesicles, this dataset could point towards a Tau KO-dependent effect on motor regulation/processivity rather than cargo binding or activation of the transport complex, which would be interesting and worth discussing.

Fig.2

It is not clear whether the S402 phosphorylation has been found in both APPswe and WT mice. In the Materials and Methods (p. 7 ) and Results (p. 13) it is mentioned that both APPswe and WT mice were used for mass spectrometry analysis although in the figures (for instance, Fig. 2D) the data showed refer to the APPswe background. Likewise, could the author make clear what mouse background was used for the IPs assays (Fig. 2B)?

- M&M: This section mentions that GSK3 inhibitor was added 30’ prior to addition of AβOs, but the main text in Results states 1 hour. Please clarify which time is the correct one. Also, please indicate whether the GSK3 inhibitor was also present during image acquisition? (This is, 18 hs after addition of AβOs, see Fig 1A).

- M&M: Please describe the composition of RIPA buffer used in immunoprecipitation experiments.

- Results: The sentence “....implying that tau-induced phosphatase signaling” would better read “ruling out tau-mediated PP1 activation”.

- Fig 2: I suggest changing the label “Ig alone” for “control IgG”.

- Fig. 2B does not provide any information and could be discarded without affecting the main conclusions in the manuscript. As documented here, MS methods are much more sensitive than anti-pSer and anti-pThr antibodies.

- Fig 3: Please clarify whether the three KIF1A constructs in this Figure correspond to human or rat versions. In addition, clarify whether this set of constructs also bear the V483N mutation that presumably abolishes auto-inhibition.

- Five papers are cited supporting the sentence: “Many studies have examined how tau hyperphosphorylation, microtubule destabilization and cytoskeletal collapse impair transport”. Although the Mandelkow et al and Stokin et al references indeed support such mechanism, the other three manuscripts don’t. Instead, those references support a different mechanism where pathogenic forms of tau, including tau phosphorylated at specific residues, inhibit axonal transport though activation of a PP1-GSK3 pathway independently of microtubule stability.

-Abstract: The abstract reads very well, but much of the final sentence is not informative. It is recommended that a final sentence summarizing the main conclusions of the work is included.

Minor points

1) It would be helpful to have molecular weight markers everywhere in the WB panels.

2) Page 14 - line 2: should Ser 932 be Ser 933 as in Fig. 2D?

3) Figure 3C, graphs. Does the -Rap treatment refers to the mutants or WT conditions.

4) The putative phosphorylation site(s) responsible for the GSK3β-mediated regulation of KIF1A transport has not been found. Therefore, I would not endorse statements such as ‘Data from our present study support the notion that the pathological activation of GSK3β can impair transport by earlier, subtler mechanisms that involve inhibitory phosphorylation of motor proteins’ (p.17).

5) It is not clear why the authors used Student’s t-tests, rather than one-way ANOVAs (which would seem more appropriate), in Fig. 1D-E, Table 1 and Fig. 3E.

8) Showing the distribution of the experimental values in Fig. 1D-E and Fig. 3E would be more informative when comparing the different conditions.

## Author Response

Dear Dr. Perlson,

Thank you very much for the reviews of our manuscript entitled “GSK3ß impairs KIF1A transport in a cellular model of Alzheimer’s disease but does not regulate motor motility at S402” by Gan et al (eN-NRS-0176-20). We are delighted and grateful that the reviewers have acknowledged the quality and impact of our work, and that you have conditionally accepted our manuscript pending revisions. Our detailed replies to specific comments (in italics) are provided below. Bold type represents textual changes introduced into the revised manuscript. Changes in the manuscript itself are denoted in red text.

Response to Reviewers’ Comments - Synthesis Statement for Author:

In absence of quantitative mass spectrometry, authors should challenge the neurons with AβOs (as in Fig. 1) or over express a constitutively active form of GSK3β (predicted to increase S402 phosphorylation and to impair transport) in KIF1A WT and S402A backgrounds. If S402 phosphorylation by GSK3β does not mediate KIF1A transport, then there should be no difference between WT and S402A backgrounds (i.e. the alanine mutant is not protective against transport reduction) and one could rule out that the effect of GSK3β on transport is via S402.

According to the reviewers’ suggestions, we have compared the motility of wildtype KIF1A and KIF1A-S402A in control and AβO-treated neurons (new data presented in Figure 3B). AβO treatment reduced anterograde flux, velocity, and run length similarly in both backgrounds, indicating that the alanine point mutation does not protect against KIF1A transport defects. This finding implies that AβO-induced overactivation of GSK3β does not regulate KIF1A motility through phosphorylation at S402A. We have modified the text as follows:

"Point mutations of S402 do not alter KIF1A motility

Does phosphorylation at S402 regulate KIF1A motility? We generated a non-phosphorylatable form of KIF1A-eGFP by inducing a Ser-to-Ala point mutation (KIF1A-S402A) at this site. Conversely, we induced a Ser-to-Glu point mutation to mimic GSK3β phosphorylation of KIF1A-eGFP at this site (KIF1A-S402E). We expressed these mutants in mouse hippocampal neurons and compared their motility to wildtype KIF1A by live cell imaging (Fig. 3A). Representative kymographs and quantification of transport parameters revealed no significant differences in flux, run length and velocity for KIF1A-S402A or KIF1A-S402E relative to wildtype KIF1A (Fig. 3A; Table 2 & 1-1). These data imply that S402 phosphorylation does not regulate KIF1A transport in hippocampal neurons. We next asked whether AβO-induced overactivation of GSK3β impairs KIF1A transport via phosphorylation at S402. We compared the motility of wildtype KIF1A-eGFP and KIF1A-S402A-eGFP in control and AβO-treated neurons (Figure 3B; Table 2 & 1-1). Kymograph analyses revealed that AβO treatment similarly reduced the anterograde fluxes, velocities and run lengths of the wildtype and mutant motors, indicating that the alanine point mutation does not protect against KIF1A transport defects. Taken together, these results indicate that GSK3β does not regulate KIF1A motility through phosphorylation at S402A.

As an alternative explanation for this outcome, endogenous wildtype KIF1A may dimerize with mutant KIF1A monomers or preferentially bind to cargo, thus masking any effects of the mutations on KIF1A motility. To investigate the effects of S402A and S402E point mutations on KIF1A motility in the absence of endogenous KIF1A transport, we performed an inducible Golgi dispersion assay ...”

Request for clarification:

Fig.1/Table 1: The difference between run lengths and fluxes needs to be clarified. In the Materials and Methods the authors define fluxes as total distance traveled by KIF1A puncta (p. 5), i.e. standardised summation of run lengths. Do they mean summation of runs instead? This is important when considering Table 1. From the analysis of KIF1A motility, it seems that the absence of Tau is generally necessary for the AβOs and inhibitor VIII-mediated effect on velocity and run length, but not on fluxes. If the fluxes do reflect the number of moving vesicles, this dataset could point towards a Tau KO-dependent effect on motor regulation/processivity rather than cargo binding or activation of the transport complex, which would be interesting and worth discussing.

We apologize for the confusion between run length and flux. To clarify, we have redefined flux as “the summation of distances traveled by all moving KIF1A puncta, standardized by the length of axon imaged and the duration of the recording”. Thus, in our experiments, flux is determined by the number of moving KIF1A puncta and by the summation of their run lengths. Because the total number of moving puncta in our “vehicle", “AβO-treated", and “inhibitor VIII-treated” experimental conditions is similar between wildtype and tau KO neurons (Table 1), the observed changes in flux are more likely caused by changes in motor processivity rather than by cargo binding or activation of the transport complex. Therefore, the reviewers are correct in their interpretation that tau may regulate KIF1A processivity. We have hypothesized on such a mechanism in the Discussion:

"Alternatively, kinesin motor activity can be inhibited by cargo detachment; in isolated squid axoplasm treated with AβOs, GSK3β phosphorylates kinesin light chain-2, promoting motor-cargo dissociation and impairing transport (Morfini et al., 2002). Interestingly, and perhaps paradoxically, we found a significant effect on KIF1A run lengths and velocity in the tau -/- neurons treated with AβOs (Fig. 1; Table 1). AβOs dysregulate a number of intracellular cascades, including those active towards tau (Cline et al., 2018). It is possible that in the absence of a tau-kinase substrate, these kinases (or phosphatases) aberrantly target regulatory sites on KIF1A that govern processivity akin to the phosphorylation of the motor domain on kinesin-1 in Drosophila (Weaver et al., 2013; Banerjee et al., 2018) and KIF5C at Ser 176 (DeBerg et al., 2013). Our study is congruent with those reports and, additionally, is first to show that GSK3β impairs KIF1A motility in a mammalian primary culture model of AD (Fig. 1).”

Fig.2: It is not clear whether the S402 phosphorylation has been found in both APPswe and WT mice. In the Materials and Methods (p. 7) and Results (p. 13) it is mentioned that both APPswe and WT mice were used for mass spectrometry analysis although in the figures (for instance, Fig. 2D) the data showed refer to the APPswe background. Likewise, could the author make clear what mouse background was used for the IPs assays (Fig. 2B)?

We apologize for the confusion on this matter. Both genotypes were used for the mass spectroscopy experiments and we have now clarified this in the figure legend. “D) Tandem mass spectroscopy on KIF1A isolated from wild type (WT) and AD model mouse (APPswe; AD) brain identified four phosphopeptides...”

For the immunoprecipitation assays performed in Figure 2, we have corrected the Materials and Methods to read: “Primary hippocampal neurons from E16 wildtype C57Bl/6 mice (Jackson Laboratory)...”

- M&M: This section mentions that GSK3 inhibitor was added 30’ prior to addition of AβOs, but the main text in Results states 1 hour. Please clarify which time is the correct one. Also, please indicate whether the GSK3 inhibitor was also present during image acquisition? (This is, 18 hs after addition of AβOs, see Fig 1A).

We apologize for this mistake. The GSK inhibitor was added 30 minutes prior to the addition of AβOs for all data presented. The sentence in the Results has been corrected to “We incubated tau+/+ and tau-/- neurons with 5 μM Inhibitor VIII (a selective, cell-permeable, competitive blocker of GSK3β; Bhat et al., 2003) for 30 min prior to AβO treatment.”

- M&M: Please describe the composition of RIPA buffer used in immunoprecipitation experiments.

Primary hippocampal neurons from E16 wildtype C57Bl/6 mice (Jackson Laboratory, Bar Harbor, ME) were lysed in ice-cold RIPA buffer (50 mM Tris; pH 7.5, 5mM EDTA, 150 mM NaCl, 1% Triton X-100) containing cOmplete protease inhibitor cocktail (Roche), Halt phosphatase inhibitor cocktail (ThermoFisher), 1 mM PMSF, and 1 mM orthovandate.

- Results: The sentence “....implying that tau-induced phosphatase signaling” would better read “ruling out tau-mediated PP1 activation”.

We have changed this sentence to read... “This effect is observed in both tau +/+ and tau -/- neurons, ruling out the possibility that tau activates PP1-GSK3β signaling to block transport, as reported previously for KIF5 in isolated squid axoplasm (Kanaan et al., 2011).”

- Fig 2: I suggest changing the label “Ig alone” for “control IgG”.

This change has been made to Figure 2.

- Fig. 2B does not provide any information and could be discarded without affecting the main conclusions in the manuscript. As documented here, MS methods are much more sensitive than anti-pSer and anti-pThr antibodies.

As suggested, we have moved Figure 2B to the extended data portion. We believe this figure is important as it highlights the challenges of using pan-phosphorylation antibodies for the detection of phosphorylation sites on KIF1A, and likely more broadly when detecting phosphoproteins. Moreover, these experiments help to explain the motivation of our strategy to pursue the more sensitive techniques such as mass spectroscopy and in vitro phosphorylation.

- Fig 3: Please clarify whether the three KIF1A constructs in this Figure correspond to human or rat versions. In addition, clarify whether this set of constructs also bear the V483N mutation that presumably abolishes auto-inhibition.

We apologize if this was not clear. In the second full paragraph of page 15, we indicate the following:

"Because full-length KIF1A resides in an inactive, autoinhibited state, we first introduced a point mutation (V483N) that relieves the self-inhibition of motor activity (Huo et al., 2012; Yue et al., 2013; Soppina et al., 2014). Using this active version of full-length KIF1A, we then introduced point mutations that could abolish or mimic the phosphorylation state of the S402 residue. Using the rat KIF1A sequence, these mutations are S411A and S411E, respectively.”

- Five papers are cited supporting the sentence: “Many studies have examined how tau hyperphosphorylation, microtubule destabilization and cytoskeletal collapse impair transport”. Although the Mandelkow et al and Stokin et al references indeed support such mechanism, the other three manuscripts don’t. Instead, those references support a different mechanism where pathogenic forms

of tau, including tau phosphorylated at specific residues, inhibit axonal transport though activation of a PP1-GSK3 pathway independently of microtubule stability.

We apologize for this mistake in the references and have removed LaPointe et al., 2009; Morfini et al., 2009, and Kanaan et al., 2011, and replaced this with one more reference to microtubule instability (Cowan et al., 2010), along with a review that discusses the current state of understanding tau in transport defects (Combs et al. 2019). The modified text now reads:

"Many studies have examined how tau hyper-phosphorylation, microtubule destabilization and cytoskeletal collapse impair transport (Mandelkow et al., 2003; Stokin and Goldstein, 2006; Cowan et al., 2010, Combs et al., 2019).”

-Abstract: The abstract reads very well, but much of the final sentence is not informative. It is recommended that a final sentence summarizing the main conclusions of the work is included.

We have modified the final sentences of our abstract to summarize the main conclusions of our work: “Finally, we tested whether a phosphomimic of S402 could modulate KIF1A motility in control and AβO-treated mouse neurons and in a Golgi dispersion assay devoid of endogenous KIF1A. In both systems, transport driven by mutant motors was similar to that of wildtype motors. In conclusion, GSK3β impairs KIF1A transport but does not regulate motor motility at S402. Further studies are required to determine the specific phosphorylation sites on KIF1A that regulate its cargo binding and/or motility in physiological and disease states.”

Minor points:

1) It would be helpful to have molecular weight markers everywhere in the WB panels.

This change has been made. Please see the revised Figure 2.

2) Page 14 - line 2: should Ser 932 be Ser 933 as in Fig. 2D?

Upon closer inspection we made a mistake in the numbering of this amino acid (GenBank BAA0622.1) and it is Ser 937. We have corrected this in the text and in Figure 2D. “...for example, Ser 937 (S937), located within the presumed cargo-binding peptide SGTS*QEELR...” We have also indicated the GenBank number in the legend of Figure 2D.

3) Figure 3C, graphs. Does the -Rap treatment refers to the mutants or WT conditions.

We apologize for the confusion. The graphs of -Rap treatment contained data pooled from both conditions since the Golgi distribution is nearly identical in the absence of rapamycin regardless of experimental condition. We now provide the data for the -Rap treatment for the WT and mutant conditions separately (new Figure 4, formerly Figure 3).

4) The putative phosphorylation site(s) responsible for the GSK3β-mediated regulation of KIF1A transport has not been found. Therefore, I would not endorse statements such as ‘Data from our present study support the notion that the pathological activation of GSK3β can impair transport by earlier, subtler mechanisms that involve inhibitory phosphorylation of motor proteins’ (p.17).

We have revised the sentence above to the following: “Data from our present study suggests, pending identification of the specific phosphorylation sites, that the pathological activation of GSK3β can impair transport by earlier, subtler mechanisms that involve inhibitory phosphorylation of motor proteins.”

5) It is not clear why the authors used Student’s t-tests, rather than one-way ANOVAs (which would seem more appropriate), in Fig. 1D-E, Table 1 and Fig. 3E.

We have now performed one-way ANOVAs followed by a Tukey’s post-hoc comparison on all data presented in figure 1, the new figure 3, and Tables 1 and 2. We also provide a table with f and p values (Table 1-1).

For the former Figure 3E (now Figure 4E), we are comparing the Golgi distribution in cells expressing the mutant KIF1A to the distribution in cells expressing the WT motor at each binned distance. Since we are comparing the means of two samples, we believe the Student’s t-test is the appropriate statistical test for the data. Nevertheless, at the reviewer’s request, we have applied an ANOVA analysis comparing all four conditions (WT -Rap, WT +Rap, mutant -Rap, and mutant +Rap) for each of the mutants and the analyses agree with results of the Student’s t-test. These analyses are provided to the reviewers as a separate Excel file.

8) Showing the distribution of the experimental values in Fig. 1D-E and Fig. 3E would be more informative when comparing the different conditions.

Please see the newly generated graphs showing the distribution of all experimental values in Figures 1 D & E and Figure 3 A & B. For the new Figure 4 (formerly Figure 3), the graphs become too “busy” if we show the distribution of experimental values and it becomes very difficult to visualize each of the curves. We thus prefer to keep these graphs as originally presented.

We appreciate the constructive critiques of the reviewers and feel that these comments have substantially improved our work. We now hope the current version of our manuscript is considered acceptable for publication in eNeuro.
