# Supplementary material for: GSK3β Impairs KIF1A Transport in a Cellular Model of Alzheimer’s Disease but Does Not Regulate Motor Motility at S402
Source: eNeuro. 2020 Nov 4;7(6):ENEURO.0176-20.2020. doi: 10.1523/ENEURO.0176-20.2020 (PMC7768277; doi:10.1523/ENEURO.0176-20.2020)
Supplement: Extended Data Table 1-1 — ANOVA outputs for trafficking values. Download Table 1-1, DOCX file. [file enu-eN-NRS-0176-20-s02.docx]

| Table 1-1 | |  |  |  |
| --- | --- | --- | --- | --- |
|  |  |  |  |  |
|  | ANOVA outputs for trafficking values | | | |
|  |  |  |  |  |
|  | Figure | Test used | Sample Size | Degrees of freedom and p-value |
|  | Fig. 1 (All Events Flux) | One-way ANOVA | 123 | F(5,117) = 17.82, p<0.0001 |
|  | Fig. 1 (All Events Velocity) | One-way ANOVA | 123 | F(5,117) = 2.737, p=0.0224 |
|  | Fig. 1 (All Events Run Length) | One-way ANOVA | 123 | F(5,117) = 6.684, p<0.0001 |
|  | Fig. 1 (Anterograde Flux) | One-way ANOVA | 123 | F(5,117) = 13.92, p<0.0001 |
|  | Fig. 1 (Anterograde Velocity) | One-way ANOVA | 123 | F(5,117) = 4.921, p=0.0004 |
|  | Fig. 1 (Anterograde Run Length) | One-way ANOVA | 123 | F(5,117) = 5.730, p<0.0001 |
|  | Fig. 1 (Retrograde Flux) | One-way ANOVA | 123 | F(5,117) = 0.5831, p=0.7129 |
|  | Fig. 1 (Retrograde Velocity) | One-way ANOVA | 123 | F(5,117) = 0.8900, p=0.4902 |
|  | Fig. 1 (Retrograde Run Length) | One-way ANOVA | 123 | F(5,117) = 1.344, p=0.2507 |
|  | Fig. 3a (All Events Flux) | One-way ANOVA | 48 | F(2,45) = 0.2192, p=0.8040 |
|  | Fig. 3a (All Events Velocity) | One-way ANOVA | 48 | F(2,45) = 0.0138, p=0.9863 |
|  | Fig. 3a (All Events Run Length) | One-way ANOVA | 48 | F(2,45) = 1.1810, p=0.3162 |
|  | Fig. 3a (Anterograde Flux) | One-way ANOVA | 48 | F(2,45) = 3.2240, p<0.0500 |
|  | Fig. 3a (Anterograde Velocity) | One-way ANOVA | 48 | F(2,45) = 0.9378, p=0.3990 |
|  | Fig. 3a (Anterograde Run Length) | One-way ANOVA | 48 | F(2,45) = 0.5003, p=0.6097 |
|  | Fig. 3a (Retrograde Flux) | One-way ANOVA | 48 | F(2,45) = 7.3860, p<0.0100 |
|  | Fig. 3a (Retrograde Velocity) | One-way ANOVA | 48 | F(2,45) = 3.5740, p<0.0500 |
|  | Fig. 3a (Retrograde Run Length) | One-way ANOVA | 48 | F(2,45) = 0.1394, p=0.8703 |
|  | Fig. 3b (All Events Flux) | One-way ANOVA | 68 | F(3,64) = 59.820, p<0.0001 |
|  | Fig. 3b (All Events Velocity) | One-way ANOVA | 68 | F(3,64) = 12.310, p<0.0001 |
|  | Fig. 3b (All Events Run Length) | One-way ANOVA | 68 | F(3,64) = 6.2320, p=0.0009 |
|  | Fig. 3b (Anterograde Flux) | One-way ANOVA | 68 | F(3,64) = 59.830, p<0.0001 |
|  | Fig. 3b (Anterograde Velocity) | One-way ANOVA | 68 | F(3,64) = 11.880, p<0.0001 |
|  | Fig. 3b (Anterograde Run Length) | One-way ANOVA | 68 | F(3,64) = 6.3670, p<0.0008 |
|  | Fig. 3b (Retrograde Flux) | One-way ANOVA | 68 | F(3,64) = 7.6540, p=0.0002 |
|  | Fig. 3b (Retrograde Velocity) | One-way ANOVA | 68 | F(3,64) = 6.4720, p=0.0007 |
|  | Fig. 3b (Retrograde Run Length) | One-way ANOVA | 68 | F(3,64) = 0.8237, p=0.4856 |
