# Supplementary figures and images for: GSK3β Impairs KIF1A Transport in a Cellular Model of Alzheimer’s Disease but Does Not Regulate Motor Motility at S402
Source: eNeuro. 2020 Nov 4;7(6):ENEURO.0176-20.2020. doi: 10.1523/ENEURO.0176-20.2020 (PMC7768277; doi:10.1523/ENEURO.0176-20.2020)

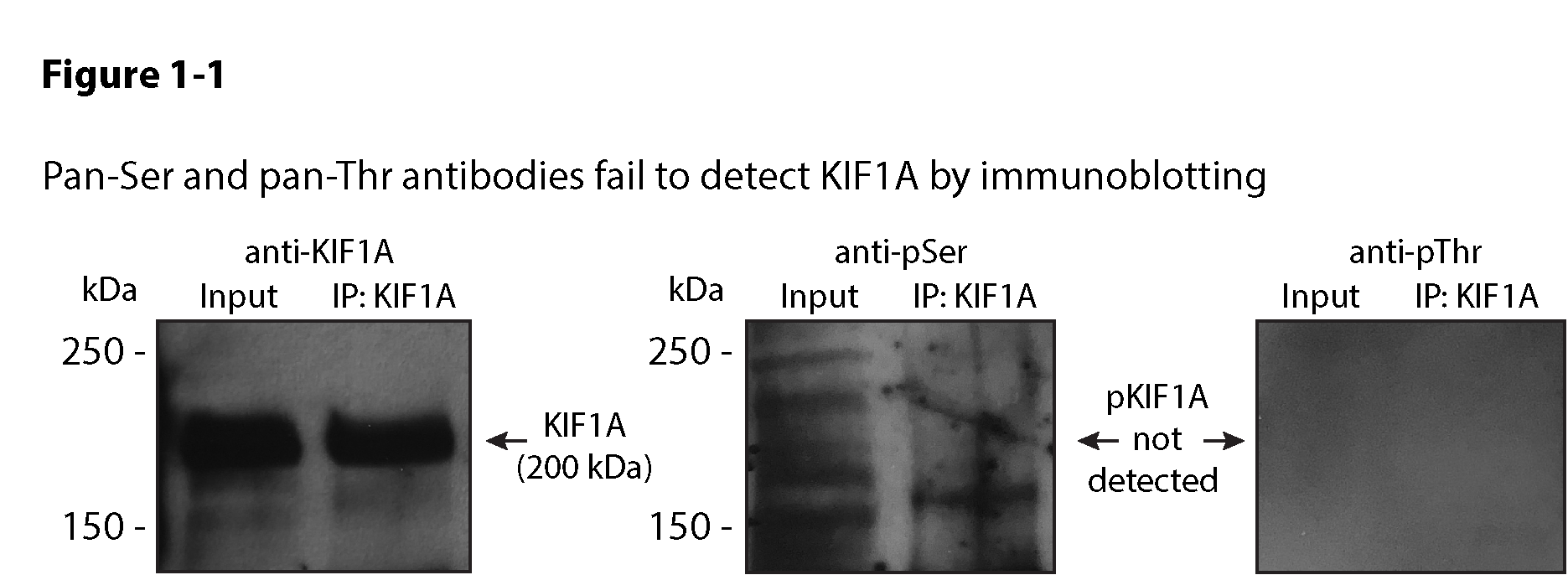

Supplement: Extended Data Figure 1-1 — Pan-serine and pan-threonine antibodies fail to detect phosphorylated KIF1A by immunoblotting in adult mouse hippocampi. Download Figure 1-1, TIF file. [file enu-eN-NRS-0176-20-s01.tif]

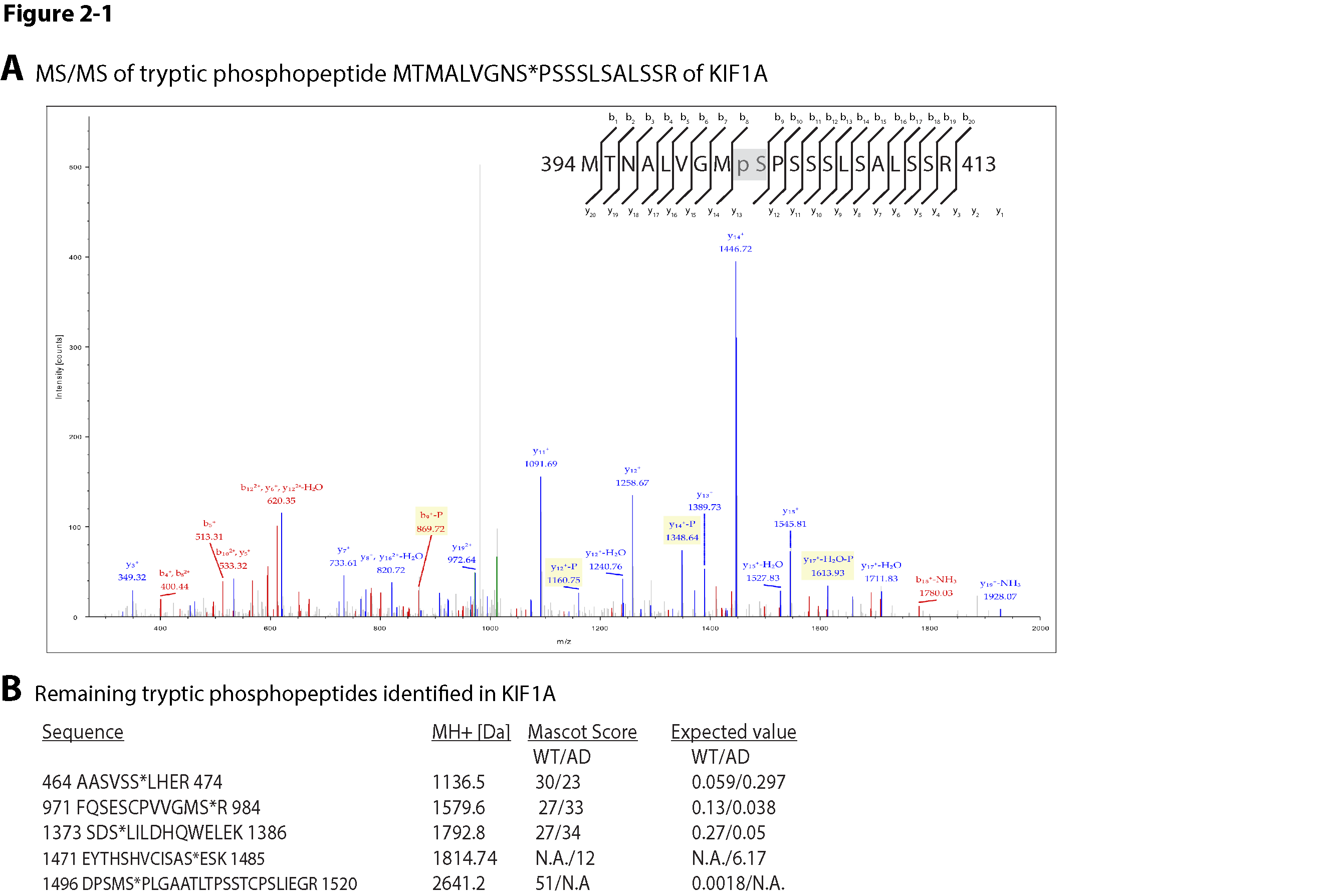

Supplement: Extended Data Figure 2-1 — Mass spectrum of the KIF1A MTMALVGNS*PSSSLSALSSR phosphopeptide. A, The graph shows the output mass spectrum, obtained from the mass spectrometer, for one of the identified phosphopeptides of the KIF1A protein. The graph plots ion intensity versus mass to charge ion ratio (M/Z) for b+ (red) and y+ (blue) ions that are the direct (N to C terminus) and reverse (C to N terminus) ion series obtained during collision-induced dissociation (CID). Also see Figure 2 in the main text. The detected ions of the b-ion and y-ion collision series are shown in the inset; *denotes phosphorylated residue. B, Identification of other phosphopeptides of KIF1A. No known kinase motifs were identified in these sequences. Download Figure 2-1, TIF file. [file enu-eN-NRS-0176-20-s03.tif]

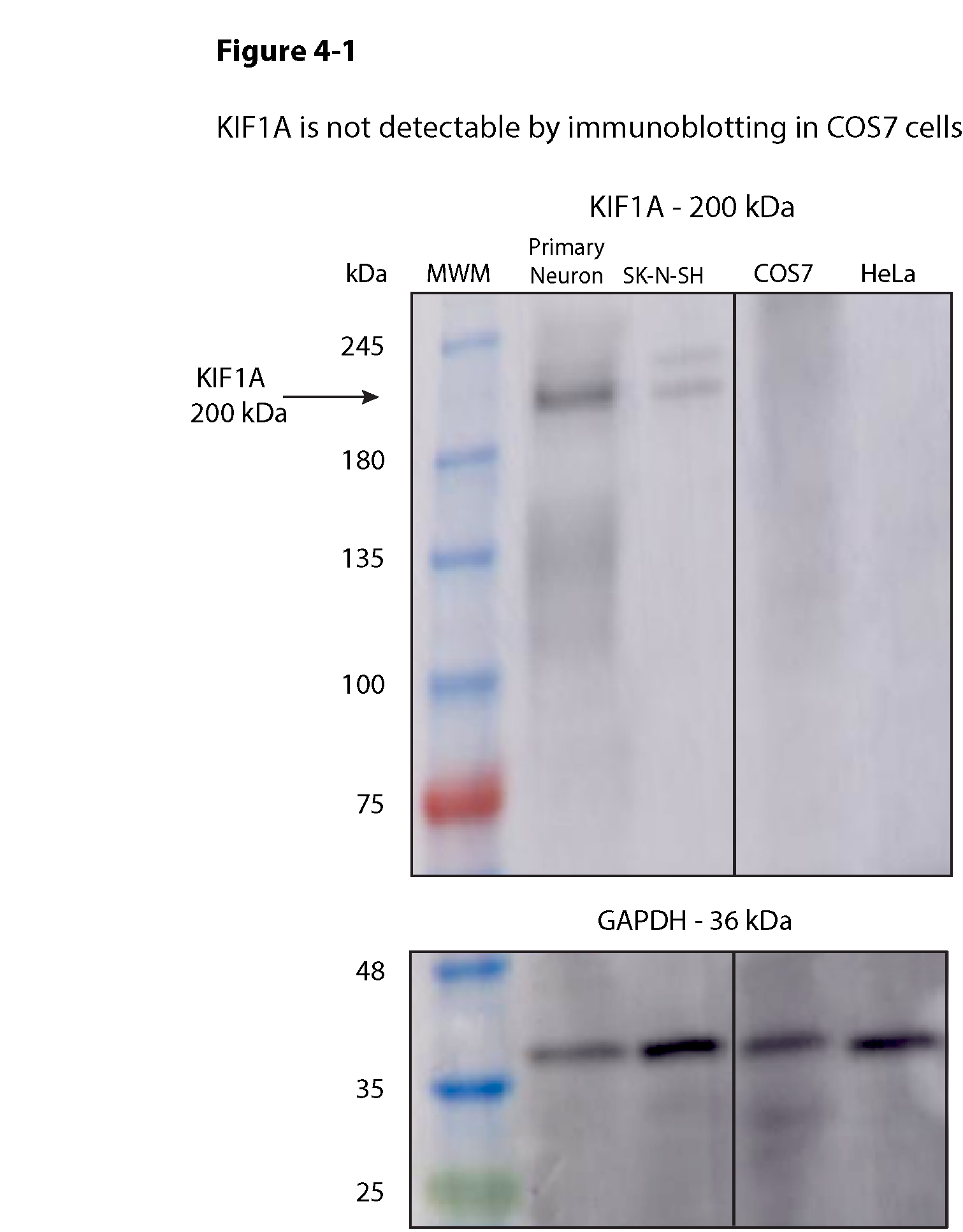

Supplement: Extended Data Figure 4-1 — KIF1A is undetectable in COS7 cells. Immunoblot analysis of endogenous KIF1A expression in neuronal and non-neuronal cells. KIF1A is detectable in primary hippocampal neurons and SK-N-SH lysates. A second band is detected in the SK-N-SH cells which may indicate the presence of a KIF1A isoform, for which at least five have been identified (https://www.ncbi.nlm.nih.gov/genbank/). Download Figure 4-1, TIF file. [file enu-eN-NRS-0176-20-s05.tif]
