# Supplementary material for: GSK3β Impairs KIF1A Transport in a Cellular Model of Alzheimer’s Disease but Does Not Regulate Motor Motility at S402
Source: eNeuro. 2020 Nov 4;7(6):ENEURO.0176-20.2020. doi: 10.1523/ENEURO.0176-20.2020 (PMC7768277; doi:10.1523/ENEURO.0176-20.2020)
Supplement: Extended Data Figure 4-2 — Student’s t test p values for Golgi dispersion assay. Download Figure 4-2, DOCX file. [file enu-eN-NRS-0176-20-s04.docx]

| Student's t test *p* values for Golgi dispersion assay | | | | | |
| --- | --- | --- | --- | --- | --- |
|  | WT vs S411A | |  | WT vs S411E | |
| Distance | - Rap | + Rap |  | - Rap | + Rap |
| 0.00 | 0.962803 | 0.044233 |  | 0.541916 | 0.067602 |
| 0.05 | 0.468823 | 0.11427 |  | 0.931064 | 0.174364 |
| 0.10 | 0.381933 | 0.407997 |  | 0.971359 | 0.292801 |
| 0.15 | 0.656459 | 0.829365 |  | 0.649796 | 0.687057 |
| 0.20 | 0.732324 | 0.529635 |  | 0.372318 | 0.592325 |
| 0.25 | 0.811037 | 0.501988 |  | 0.210185 | 0.514569 |
| 0.30 | 0.992751 | 0.944002 |  | 0.140102 | 0.780682 |
| 0.35 | 0.501951 | 0.548918 |  | 0.487385 | 0.244327 |
| 0.40 | 0.894184 | 0.432136 |  | 0.90704 | 0.096072 |
| 0.45 | 0.453295 | 0.275786 |  | 0.466388 | 0.071954 |
| 0.50 | 0.483354 | 0.126238 |  | 0.306621 | 0.05886 |
| 0.55 | 0.854281 | 0.090064 |  | 0.343756 | 0.124569 |
| 0.60 | 0.811921 | 0.185783 |  | 0.613846 | 0.140604 |
| 0.65 | 0.703655 | 0.379384 |  | 0.774936 | 0.60354 |
| 0.70 | 0.215695 | 0.897443 |  | 0.731036 | 0.907608 |
| 0.75 | 0.206487 | 0.956263 |  | 0.339551 | 0.998534 |
| 0.80 | 0.260352 | 0.721923 |  | 0.483078 | 0.55522 |
| 0.85 | 0.535792 | 0.722131 |  | 0.93352 | 0.932527 |
| 0.90 | 0.510656 | 0.993625 |  | 0.734436 | 0.461218 |
| 0.95 | 0.890038 | 0.723478 |  | 0.441587 | 0.879024 |
| 1.00 | 0.651897 | 0.784528 |  | 0.063153 | 0.475833 |

Table 4-1
